# Genome-Wide association study identifies candidate genes for Parkinson's disease in an Ashkenazi Jewish population

**DOI:** 10.1186/1471-2350-12-104

**Published:** 2011-08-03

**Authors:** Xinmin Liu, Rong Cheng, Miguel Verbitsky, Sergey Kisselev, Andrew Browne, Helen Mejia-Sanatana, Elan D Louis, Lucien J Cote, Howard Andrews, Cheryl Waters, Blair Ford, Steven Frucht, Stanley Fahn, Karen Marder, Lorraine N Clark, Joseph H Lee

**Affiliations:** 1 Taub Institute for Research on Alzheimer's Disease and the Aging Brain, College of Physicians and Surgeons, Columbia University, New York, NY, USA; 2 Gertrude H. Sergievsky Center, College of Physicians and Surgeons, Columbia University, New York, NY, USA; 3 Department of Pathology and Cell Biology, College of Physicians and Surgeons, Columbia University, New York, NY, USA; 4 Department of Neurology, College of Physicians and Surgeons, Columbia University, New York, NY, USA; 5 Department of Epidemiology, Mailman School of Public Health, Columbia University, New York, NY, USA; 6 New York State Psychiatric Institute, Data Coordinating Center, New York, NY, USA; 7 Department of Psychiatry, Columbia University Medical Center, NYC, NY, USA; 8 Center for Human Genetics, College of Physicians and Surgeons, Columbia University, New York, NY, USA

**Keywords:** Parkinson's disease, GWAS, Ashkenazi Jews, case-control study, candidate genes

## Abstract

**Background:**

To date, nine Parkinson disease (PD) genome-wide association studies in North American, European and Asian populations have been published. The majority of studies have confirmed the association of the previously identified genetic risk factors, *SNCA *and *MAPT*, and two studies have identified three new PD susceptibility loci/genes (*PARK16, BST1 *and *HLA-DRB5*). In a recent meta-analysis of datasets from five of the published PD GWAS an additional 6 novel candidate genes (*SYT11, ACMSD, STK39, MCCC1/LAMP3, GAK *and *CCDC62/HIP1R*) were identified. Collectively the associations identified in these GWAS account for only a small proportion of the estimated total heritability of PD suggesting that an 'unknown' component of the genetic architecture of PD remains to be identified.

**Methods:**

We applied a GWAS approach to a relatively homogeneous Ashkenazi Jewish (AJ) population from New York to search for both 'rare' and 'common' genetic variants that confer risk of PD by examining any SNPs with allele frequencies exceeding 2%. We have focused on a genetic isolate, the AJ population, as a discovery dataset since this cohort has a higher sharing of genetic background and historically experienced a significant bottleneck. We also conducted a replication study using two publicly available datasets from dbGaP. The joint analysis dataset had a combined sample size of 2,050 cases and 1,836 controls.

**Results:**

We identified the top 57 SNPs showing the strongest evidence of association in the AJ dataset (p < 9.9 × 10^-5^). Six SNPs located within gene regions had positive signals in at least one other independent dbGaP dataset: *LOC100505836 *(Chr3p24), *LOC153328*/*SLC25A48 *(Chr5q31.1), *UNC13B *(9p13.3), *SLCO3A1*(15q26.1), *WNT3*(17q21.3) and *NSF *(17q21.3). We also replicated published associations for the gene regions *SNCA *(Chr4q21; rs3775442, p = 0.037), *PARK16 *(Chr1q32.1; rs823114 (*NUCKS1*), p = 6.12 × 10^-4^), *BST1 *(Chr4p15; rs12502586, p = 0.027), *STK39 *(Chr2q24.3; rs3754775, p = 0.005), and *LAMP3 *(Chr3; rs12493050, p = 0.005) in addition to the two most common PD susceptibility genes in the AJ population *LRRK2 *(Chr12q12; rs34637584, p = 1.56 × 10^-4^) and *GBA *(Chr1q21; rs2990245, p = 0.015).

**Conclusions:**

We have demonstrated the utility of the AJ dataset in PD candidate gene and SNP discovery both by replication in dbGaP datasets with a larger sample size and by replicating association of previously identified PD susceptibility genes. Our GWAS study has identified candidate gene regions for PD that are implicated in neuronal signalling and the dopamine pathway.

## Background

Genetic linkage studies of Parkinson's Disease (PD) have identified susceptibility loci in five genes which include *SNCA *[1] (*PARK1*), *Parkin *[2] (*PARK2*), PTEN-induced putative kinase [3] (*PINK1*;*PARK6*), *DJ-1 *[4] (*PARK7*) and Leucine rich repeat kinase 2 [5] (*LRRK2*; *PARK8*). Mutations in these genes are rare and highly penetrant with large effects (e.g *Parkin, PARK2*), and their prevalence may vary substantially by age at onset (AAO), family history of PD (FHPD), and ethnicity [6,7]. On the other hand, common genetic variants defined as variants with a minimum allele frequency (MAF) of 5% to 20-30% are also believed to contribute to PD disease susceptibility. Often genome wide association studies (GWAS) exclude SNPs with low allele frequencies (MAF < 5%), thereby excluding some rare variants that may contribute to disease susceptibility. To date, nine PD GWAS studies in North American, European and Asian populations have been published [8-16]. While the majority of studies have confirmed the association of the previously identified genetic risk factors, *SNCA *and *MAPT*, only two studies have identified three new PD susceptibility genes that reached genome wide significance [13,16]. In a Japanese population, a GWAS identified the new susceptibility loci *PARK16 *at chr1q32 and *BST1 *on 4p15 and the HLA region was identified as a susceptibility locus in a late-onset sporadic PD population from North America [13,16]. In a recent meta-analysis of datasets from five of the published PD GWAS an additional 6 novel candidate genes (*SYT11, ACMSD, STK39, MCCC1/LAMP3, GAK *and *CCDC62/HIP1R*) were identified [17]. Collectively the associations identified in these GWAS account for only a small proportion of the estimated total heritability of PD suggesting that an 'unknown' component of the genetic architecture of PD remains to be identified. Some of the contributing factors to the difficulties in identification of risk variants are: etiologic heterogeneity across populations, other genetic mechanisms like methylation, and the importance of multiple, rare variants in common diseases, which are not well captured by current GWAS approaches.

To overcome some of these limitations, we applied a GWAS approach to a relatively homogeneous Ashkenazi Jewish (AJ) population living in the New York area to search for both 'rare' and 'common' genetic variants that confer risk of PD by examining any SNPs with allele frequencies exceeding 2%. We have focused on a genetic isolate, the AJ population, as a discovery dataset since this cohort has a higher sharing of genetic background, and historically experienced a significant bottleneck, thereby potentially increasing allele frequencies such that some rare variants in other European populations may be more frequent in the AJ population [18-23].

Our study had three main aims. First, we used our AJ case-control population as a discovery dataset and performed a GWAS using an overall MAF threshold of > 2% to identify novel candidate SNPs, and conducted a replication study using two publicly available datasets from dbGaP (CIDR/Pankratz et al 2009 [24] Genome wide association study in familial PD and NINDS: The National Institute of Neurological Disorders and Stroke [11]). Second, we re-analyzed the dbGaP datasets from CIDR/Pankratz et al 2009 and NINDS using an overall MAF threshold of 2% or higher to identify rare genetic variants in these datasets, and attempted to replicate the findings in the AJ dataset. Third, we examined susceptibility and candidate genes identified in previously published GWAS and other association studies, including *MAPT, SNCA, LRRK2, GBA, PARK16, BST1, HLA-DRA, SYT11, ACMSD, STK39, MCCC1/LAMP3, GAK*, and *CCDC62/HIP1R *in all three datasets. While the sample size of the AJ discovery set is relatively small, the joint analysis dataset had a combined sample size of 2,050 cases and 1,836 controls.

## Methods

### Subjects

The AJ GWAS dataset was created by combining participants from two studies the Genetic Epidemiology of PD study (PD EPI) and the AJ Study. The ascertainment of cases (n = 168) and controls (n = 84) for the PD EPI study was described in detail in Marder et al. [25] and the ascertainment of cases (n = 100) and controls (n = 94) for the AJ study is described below. Briefly, for the PD EPI and AJ study, PD cases were recruited from the Center for PD and Other Movement Disorders at Columbia University. All met research criteria for PD. All controls underwent the same evaluation as cases, which included a medical history, Unified Parkinson's Disease Rating Scale (UPDRS) and Mini Mental State Exam (MMSE). Family history of PD and related disorders in first-degree relatives was obtained using a structured interview that has been shown to be reliable and valid.

The PD EPI study was enriched for cases with AAO of 50 years of age or younger and the majority of controls were recruited via random digit dialling. Information on Jewish ancestry in each of the grandparents was obtained during an interview. Information about Ashkenazi origin was not specifically obtained; however ~90% of Jews in the United States are Ashkenazi. For the AJ study, PD cases were recruited specifically based on their AJ ancestry and information on Ashkenazi Jewish ancestry in each of the grandparents was obtained during an interview. This study was approved by the Institutional Review Board at Columbia University Medical Center. Each study participant signed a written informed consent approved by the University Human Ethics Committee.

### Genotyping and Quality Control Assessment

A total of 268 cases and 178 controls were genotyped using the Illumina Human 610-quad bead arrays (Cases n = 91 and Controls n = 96) or the Illumina Human 660-quad bead arrays (Cases n = 191 and Controls n = 84). All DNAs were derived from whole blood.

**Figure 1:**
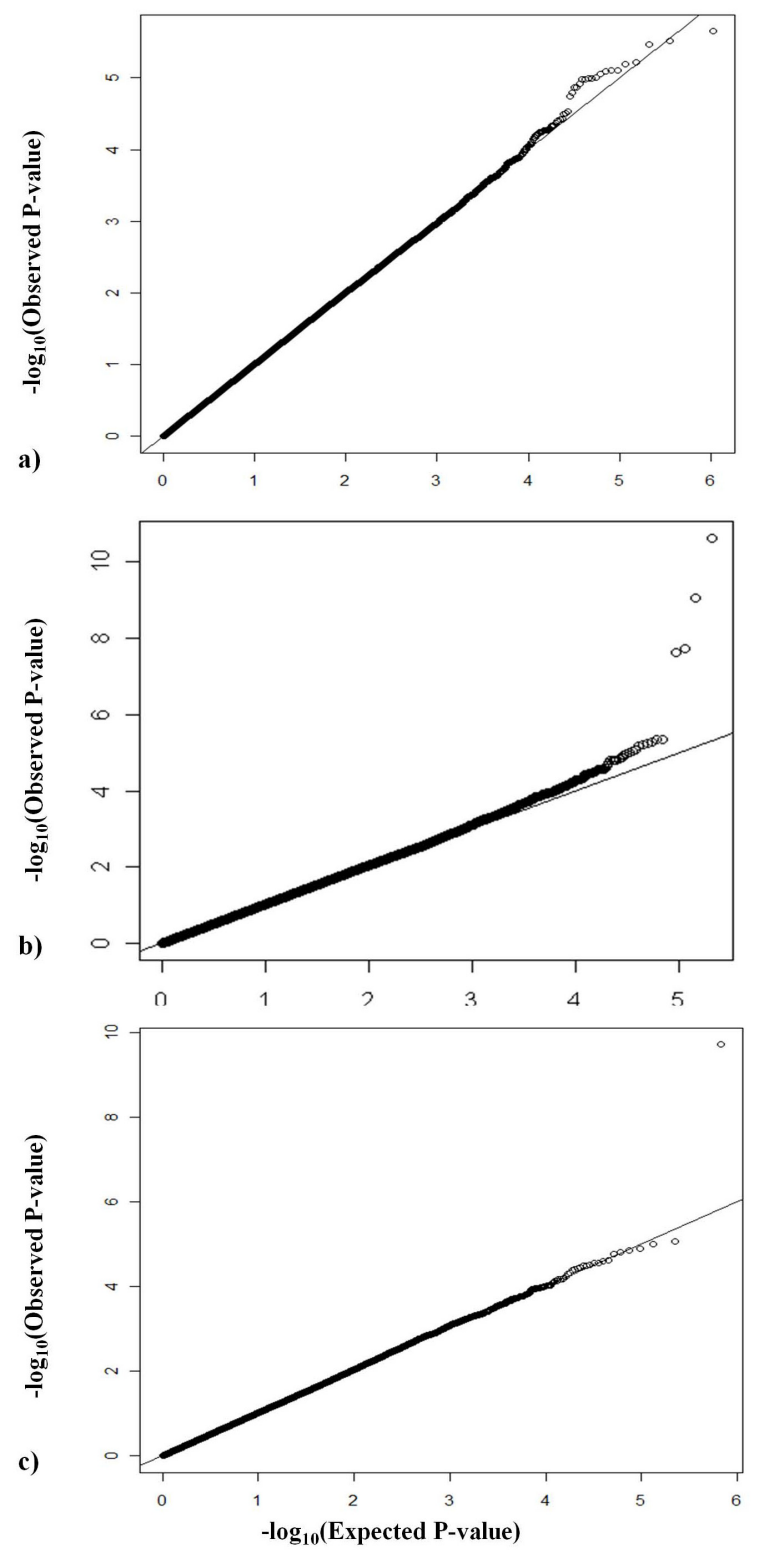
**Quantile-Quantile plots for test statistics for SNPs passing quality control in each dataset**. Figure shows -log_10 _(p-value) of observed association statistics in Y-axis, compared to -log_10 _(p-value) of the association statistics expected under the null hypothesis of no association in X-axis. The solid line represents concordance of observed and expected values. The genomic inflation factor, λ, is shown for each dataset. A) Ashkenazi Jewish (λ = 1.003), b) NINDS (λ = 1.026), and c) CIDR/Pankratz et al 2009(λ = 1.031).

Quality scores were determined from allele cluster definitions for each SNP as determined by the Illumina GenomeStudio Genotyping Module version 3.0 and the combined intensity data from 100% of study samples. Genotype calls with a quality score (Gencall value) of 0.25 or higher were considered acceptable. We genotyped 10 samples in duplicate to assess genotyping accuracy and found blind duplicate reproducibility to be 100%. In addition, 10 cases and 2 controls were genotyped by the above 2 platforms, and for the overlapping SNPs, genotypes matched. 6 individuals was removed with similar genotype with others in the IBD analysis using PLINK http://pngu.mgh.harvard.edu/~purcell/plink/ [26]. Subsequently, for the samples with duplicates, we used the Illumina Human 660-quad bead arrays. Overall we performed additional quality control (QC) measures using PLINK. We excluded SNPs with the following characteristics: missing genotyping rate > 5%; minimum allele frequency < 2%; Hardy-Weinberg Equilibrium (HWE) test [27] at a p-value < 0.0001 in controls. This screen reduced the total number of analyzed SNPs by 1.67%. Following all QC measures, we analyzed 525,124 SNPs. Figure 1a) represents the Q-Q plot for the AJ dataset. Q-Q plot was generated using the WGAviewer program [28]. SNAP http://broad.mit.edu/mpg/snap/ was used to identify and to annotate nearby SNPs in linkage disequilibrium (proxies) based on HapMap, to query and display LD and regional association plot with GWAS results [29].

*GBA *and *LRRK2 *mutation status have been reported previously in the AJ sample derived from PD EPI study previously [22,30]

### Population stratification

We examined ancestry for each subject to estimate cryptic population stratification using the identity-by-state (IBS) based clustering method as implemented in PLINK [26]. Briefly, we used all available SNPs (n = 522,578 autosomal SNPs) for the PLINK analysis (version 1.05) to assess underlying population structure. To assess potential cryptic population stratification, we augmented the 446 AJ samples with white subjects from the HapMap website http://www.hapmap.org/, which included 60 European Americans, 60 Yorubans and 90 Asians. The best fitting model assumed two underlying populations; however, the proportion of the second cluster was small (n = 14), and this group of individuals clustered with the HapMap whites. These subjects were not dropped from the analysis.

### Statistical Analysis

We conducted single point allelic association analysis using the Mantel-Haenszel chi-squared test statistic, which tests for SNP-disease association conditional on population sub-cluster estimated from the PLINK analysis described above (Additional file 1). For the SNPs with the strongest support for association and are located within a gene, we performed two additional analyses for the region containing the top SNPs and for several PD genes that have been previously reported. First, we conducted haplotype analysis using 2 or 3 contiguous SNPs as implemented in the PLINK program. This approach computed a Wald statistic p value for comparing each haplotype between cases and controls as well as an overall p-value which compares the frequencies across all the haplotypes [26]. Second, we performed odds ratios adjusting for age and sex as implemented in PLINK (Results; Additional files 1 and 2).

### Candidate Gene Analyses

We performed separate analyses focusing on SNPs in the candidate genes that were identified from previous genome wide association studies, including *MAPT, SNCA, LRRK2, GBA, PARK16, BST1, HLA-DRA, SYT11, ACMSD, STK39, MCCC1/LAMP3, GAK and CCDC62/HIP1R*. For these genes, we computed Mantel-Haenzel chi-squared test to assess allelic association and computed odds ratios adjusting for sex and age to assess the effect size of the SNP in the AJ population.

### Replication Datasets

To determine whether the findings from the Ashkenazi Jewish discovery samples are supported in independent samples, we examined the publicly available GWAS data for the CIDR/Pankratz et al 2009 [24] and NINDS PD GWAS [11]http://www.ncbi.nlm.nih.gov/sites/entrez?db=gap; Downloaded on September 25th, 2009. NINDS dataset comprise 931 cases and 798 controls, while the CIDR/Pankratz et al 2009 dataset comprise 900 cases and 867 controls. After applying the same QC measures to these two datasets as those applied to the AJ dataset, the final datasets included 923 cases and 798 controls for the NINDS dataset, and 859 cases and 860 controls for the CIDR/Pankratz et al 2009 dataset. Characteristics of the two datasets are described in Table 1.

**Table 1:** Characteristics of the genotyped subjects from the Ashkenazi Jewish, NINDS, and CIDR/Pankratz et al 2009 datasets

	Ashkenazi Jews		NINDS		CIDR/Pankratz et al 2009		Overall	
	
	Case	Control	Case	Control	Case	Control	Case	Control
Subjects (%)	268(60.1)	178(39.9)	923(53.6)	798(46.4)	859(50.0)	860(50.0)	2050(52.8)	1836(47.2)

Mean age at onset/examination Year ± SD*	59.9 ± 12.1	69.8 ± 8.8	58.5 ± 13.2	58.6 ± 16.4	61.8 ± 10.9	54.8 ± 13.0	60.0 ± 12.2	57.9 ± 14.9

Male(%)	255(57.2)		886(51.5)		850(49.4)		1991(51.2)	

Proportion of EOPD^#^	24.5%		26.0%		15.0%		21.3%	

Total	446		1721		1719		3886	

Genotyping rate	99.85		99.79		99.81		99.82	

Markers passing QC	525124		517805		334161			

* Age at onset for cases and age at examination for controls, ^# ^EOPD: Early Onset of PD, with age at onset younger than or equal to 50

We applied the same allelic association model to the replication datasets to determine whether the candidate SNPs from the AJ dataset are associated with PD in these two unrelated datasets. To assess the overall effect of candidate SNPs, we then conducted a meta-analysis using the weighted Z-score meta-analysis as implemented in METAL (http://www.sph.umich.edu/csg/abecasis/metal/). For some SNPs, only two of three datasets were used because the SNPs were not available in all datasets and imputation was unreliable.

## Results and discussion

Table 1 shows the summary characteristics of genotyped subjects for the AJ discovery dataset and two replication datasets of white subjects that were comparable in demographic and clinical characteristics. The AJ dataset had a slightly larger number of cases (n = 268) versus controls (n = 178), but the remaining two datasets had a comparable number of cases versus controls. To control for population stratification, we excluded subjects who clustered differently from the majority of Ashkenazi Jews (See Subsection Population Stratification, Materials and Methods section for details).

Overall, the NINDS dataset obtained from the dbGaP consisted of 931 PD cases and 798 controls, a total of eight PD cases were excluded because these individuals were missing genotype data from the Human Hap300v1. The CIDR/Pankratz et al 2009 dataset consisted of 900 cases and 867 controls. We excluded 41 cases and seven controls with genotyping rates < 99%. These two datasets were downloaded from the dbGaP site on September 25th, 2009; therefore, the number of cases and controls do not necessarily agree with the publications for the two studies [11,24]. For these two external datasets, we did not have information on the AJ background for each subject. In these three datasets, the proportion of male subjects was slightly higher in the AJ dataset than in the two other datasets. The mean age at onset for the affected individuals in the AJ dataset was 59.9 and was comparable to the affecteds in the NINDS dataset (58.5 years) and the CIDR/Pankratz et al 2009 dataset (61.8). The proportion of early onset PD (defined as onset ≤50 years of age) was comparable between the AJ- and NINDS datasets (Table 1). The mean age at examination for unaffected individuals for the AJ dataset was older (69.8 years) than the controls in the replication datasets (58.6 for the NINDS dataset, and 54.8 for the CIDR/Pankratz et al 2009 dataset).

### Genome Wide and Allelic Association Study Results

#### AJ dataset

**Figure 2:**
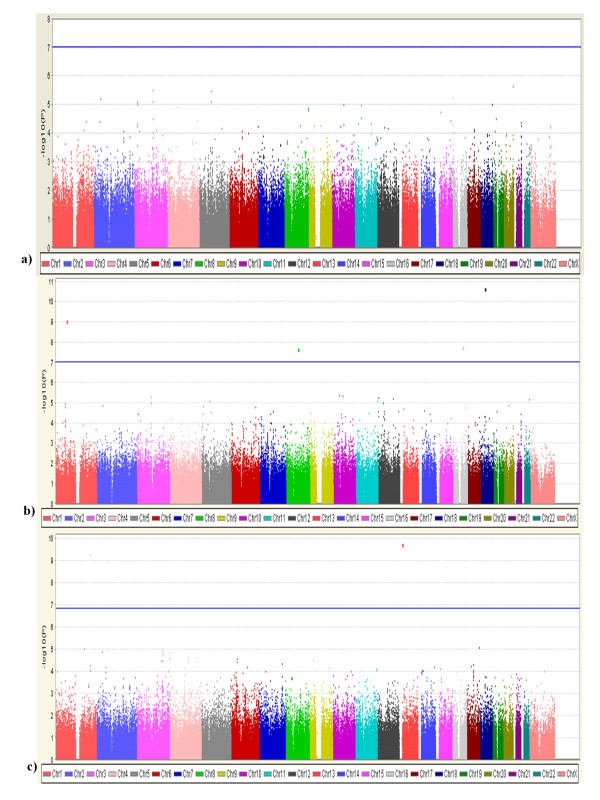
**Manhattan plot for results of GWAS. a) Ashkenazi Jewish, b) NINDS and c) CIDR/Pankratz et al 2009**. Manhattan plot of GWAS results of testing for association with PD. Horizontal axis is the genomic position, and vertical axis is -log_10 _(p-value) in 3 datasets. Blue line indicates the threshold of genome-wide significance level (Ashkenazi Jewish, p = 9.5**×**10^-8^; NINDS, p = 9.7**×**10^-8^; CIDR/Pankratz et al 2009, p = 1.5**×**10^-7^).

**Figure 3:**
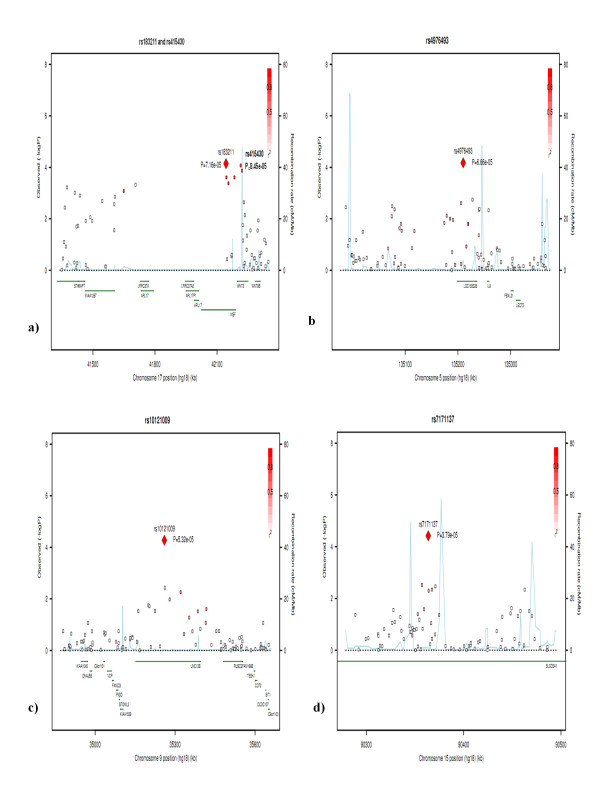
**Regional Association Plots for SNPs significant in the association analysis in the Ashkenazi Jewish dataset a) *NSF *and *WNT3*, b) *LOC153228/SLC25A48*, c) *UNC13B*, d) *SLCO3A1***. It shows -log_10 _(p-value) from association analysis for all SNPs in the region around the top SNP (surrounding 1000 kb total). X-axis shows position of the SNPs along chromosome; Y-axis gives -log_10_(p-value). P-values were obtained from GWAS in each dataset. Y-axis in the right shows recombination rate (cM/Mb). r^2 ^shows measure of the LD between this SNP and target SNP.

We identified seven candidate SNPs of high priority from the AJ discovery dataset (Table 2). Specifically, we identified the top 57 SNPs with P value < 9.9 × 10^-5 ^from the AJ discovery dataset (Additional file 1; Figure 2a). Although these SNPs do not meet the stringent Bonferroni corrected genome wide significance p-value of 9.5 × 10^-8^, these SNPs provide the strongest support for harbouring susceptibility genes for PD. To further screen these 57 candidate SNPs, we checked to see whether they were: (1) located within genic regions, and (2) replicated in at least one independent dataset. Twenty-seven out of 57 SNPs were located in or near to genes (Additional file 1), and the remainder of SNPs were located in intergenic regions. When we evaluated those SNPs in the two replication data sets, we identified six SNPs which were located within six candidate genes, namely *LOC100505836, LOC153328/SLC25A48, UNC13B, SLCO3A1, WNT3*, and *NSF *(Table 2 Figure 3). Of the six SNPs located within a gene, for three SNPs (rs10121009, rs7171137, and rs183211), the direction of allelic association was the same in all three datasets, whereas for SNPs rs415430, rs4976493 and rs1694037 the direction was the same in two datasets.

**Table 2:** Top Candidate SNPs from the Ashkenazi Jewish, NINDS and CIDR/Pankratz et al 2009 Datasets

					Ashkenazi Jewish			NINDS			CIDR/Pankratz et al 2009			Meta-analysis			
					
SNP	CHR	BP	A1	GENE	F_A	P	OR	F_A	P	OR	F_A	P	OR	A2	Z	P	Direction
					
					F_U		95% CI	F_U		95% CI	F_U		95% CI		score		
rs1694037	3	21941114	T	*LOC10055836*	0.096	1.02 × 10^-5^	0.42	0.097	0.849	1.02	0.121	0.049	1.24	T	-1.3	0.193	**- ++**
					0.199		0.29-0.62	0.095		0.81-1.28	0.100		1.00-1.54				

rs4976493	5	135210022	A	*SLC25A48*	0.442	6.66 × 10^-5^	1.77	0.403	0.026	1.17	0.367	0.190	0.91	A	2.8	0.005	**++-**
					0.309		1.34-2.35	0.366		1.02-1.34	0.389		0.79-1.05				

rs10121009	9	35259819	A	*UNC13B*	0.131	5.32 × 10^-5^	0.49	0.182	0.045	0.84	0.185	0.032	0.83	A	-4.7	2.75 × 10^-6^	**- - -**
					0.235		0.34-0.69	0.209		0.71-1.00	0.214		0.70-0.98				

rs4745122*	9	73582507	A	Intergenic	0.351	5.48 × 10^-5^	1.87	0.243	0.007	1.25	0.234	0.748	0.97	A	3.7	2.15 × 10^-4^	**++-**
					0.225		1.38-2.54	0.205		1.06-1.47	0.239		0.83-1.14				

rs7171137	15	90363631	A	*SLCO3A1*	0.397	3.79 × 10^-5^	1.84	0.370	0.013	1.20	0.351	0.620	1.04	A	4.1	4.09 × 10^-5^	**+++**
					0.264		1.38-2.47	0.330		1.04-1.38	0.343		0.90-1.19				

rs183211	17	42143493	T	*NSF*	0.207	7.16 × 10^-5^	0.54	0.213	0.102	0.87	0.196	0.001	0.77	T	-5.1	2.80 × 10^-7^	**- - -**
					0.326		0.40-0.73	0.236		0.75-1.03	0.241		0.65-0.90				

rs415430	17	42214305	C	*WNT3*	0.202	8.45 × 10^-5^	0.54	0.173	0.002	0.77				C	-5.0	6.87 × 10^-7^	--
--					0.317		0.40-0.74	0.214		0.65-0.91							

A1: Minor allele, A2: Associated allele, F_A: Minor allele freq. of case, F_U: Minor allele freq. of control, OR: odds ratios, 95% CI: 95% confidence interval, * rs4745122 is located ~8.9Kb proximal to *TMEM2*.

#### NINDS and CIDR/PANKRATZ

In the NINDS Dataset, we re-examined the data set and identified four SNPs that reached genome wide significance at p < 9.7 × 10^-8 ^(Figure 2b, Additional file 2). Of the four SNPs, one SNP (rs3784847) was located within a gene, *CDH8*, and the remaining three SNPs were in intergenic regions. The allele frequencies were low for these SNPs (< 10%). In the CIDR/Pankratz et al 2009 dataset, we identified one SNP (rs2451078) that reached genome-wide significance with p < 1.94 × 10^-10 ^(Figure 2c, Additional file 2), and this SNP (rs2451078) was located in an intron of the gene transmembrane phosphoinositide 3-phosphatase and tensin homolog 2 (*TPTE2*). SNPs that reached genome wide significance in the NINDS and CIDR/Pankratz et al 2009 datasets were not replicated in the AJ or a second dataset (data not shown) and thus we did not pursue further.

### SNP and Haplotype Analysis for Candidate Loci

#### *LOC153328*/*SLC25A48 *(5q31.1)

Rs4976493 in *LOC153328*/*SLC25A48 *was associated with PD in the AJ and NINDS datasets, but not in the CIDR/Pankratz et al 2009 dataset. However, the meta-analysis based on the three datasets supported association with PD (rs4976493, p = 0.005) (Table 2). We then performed a 2-mer and 3-mer sliding window haplotype analysis (Additional file 3a). Strongest association in the AJ dataset was observed for a 2-mer haplotype rs4976493- rs4246802 ('GG') (AJ: p = 6.93 × 10^-5^; NINDS: p = 0.025) (Additional file 3a). In the NINDS dataset, the haplotype involving rs2304075-rs6596270 'TT' was most significantly associated with PD (p = 0.008, with global-p = 0.027) and this haplotype was also significant in the AJ dataset (p = 0.003, with global-p = 0.004).

#### *UNC13B *(9p13.3)

rs10121009 was consistently associated with PD in all three datasets (Table 2 meta analysis p = 2.75 × 10^-6^) and the direction of association was consistent across studies. Our 2-mer and 3-mer sliding window haplotype analysis identified strongest association for a 3-mer haplotype 'rs7040048- rs10121009- rs10114937 ('AAG') (AJ: p = 5.09 × 10^-5^; NINDS: p = 0.046; CIDR/Pankratz et al 2009: p = 0.035) (Additional file 3b) in each dataset.

#### *SLCO3A1 *(15q26.1)

Allele A in rs7171137 was consistently associated with increased risk of PD in all the AJ and NINDS datasets and the meta analysis supported the association (p = 4.09 × 10^-5^, Table 2). Moreover, haplotype 'GA' at SNPs rs2387400-rs7171137 was associated with PD (p = 4.04 × 10^-5 ^in the AJ dataset and 0.014 in the NINDS dataset) (Additional file 3c).

#### *NSF *(17q21.31) and *WNT3 *(17q21.32)

**Figure 4:**
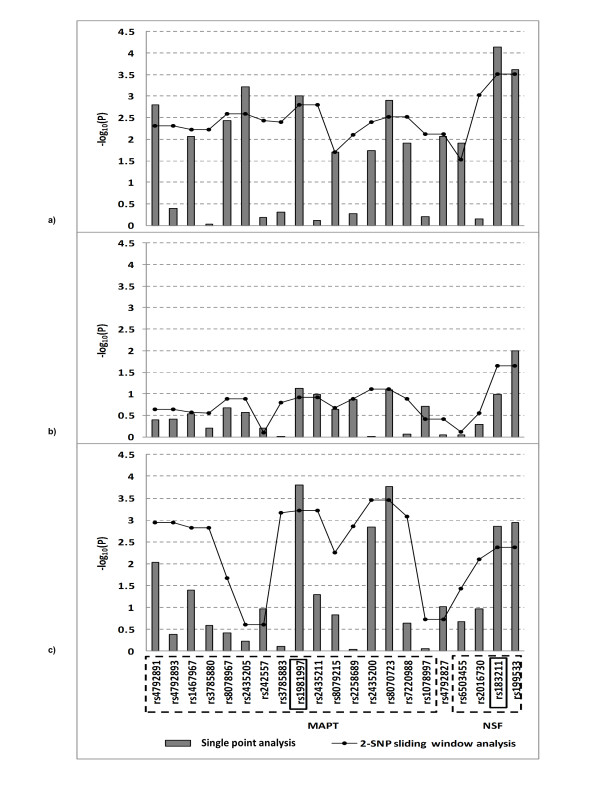
**Single point and haplotype analysis of *MAPT-NSF *region in a) Ashkenazi Jewish, b) NINDS and c) CIDR/Pankratz et al 2009 datasets**. 2-SNP sliding window analysis the strongest p-value was present from the exhaustive search.

Because the two genes are closely located with each other and with *MAPT*, we present each gene independently first, then the region as a whole, including *MAPT*. We observed a strong single and haplotype association between PD and rs183211 (*NSF*) in the AJ and CIDR/Pankratz et al 2009 datasets, but not in the NINDS dataset (Figure 4a, b, c). In addition, *WNT3*, located adjacent to NSF was also associated with PD in the AJ and NINDS datasets (Figure 3a, Table 2 Additional file 3d). In comparison, rs1981997, a SNP with the strongest support in *MAPT*, was similarly, but slightly weakly, associated with PD in the AJ dataset (p = 0.0009) and the CIDR/PANKRATZ dataset (p = 0.0002).

Because *NSF *and *WNT3 *are closely located to *MAPT *and multiple datasets show support for possible association with PD, we examined this region encompassing *MAPT-NSF-WNT3 *further. For this purpose, we used the three most significant SNPs for each gene: (rs183211) in *NSF*, one 'H1 TagSNP' (rs1981997) in *MAPT*, and rs415430 in *WNT3*. This analysis is to determine whether the *NSF *and *WNT3 *SNP confer an independent  association or whether the association with the NSF SNP was primarily due to high linkage disequilibrium between the two SNPs. We reasoned that, if there exist an independent contribution from *NSF, WNT3*, or both, we expect to see allelic or haplotype association in *NSF, WNT3 *or both, regardless of the SNP allele at *MAPT*. However, this analysis is limited in the present study, because the allele frequency of the 'A' allele is low in all three datasets (i.e., 0.216 in the AJ, 0.202 in the NINDS, and 0.202 in the CIDR/PANKRANTZ dataset). Table 3 supports that the C-T haplotype at *NSF *and *WNT3 *was associated with PD (p = 1.91 × 10^-5^). When we extended the analysis to include the 3-mer haplotypes by including the associated SNP at *MAPT*, we observed that G-C-T haplotype was strongly associated with PD as was the C-T haplotype at *NSF *and *WNT3 *(p = 0.00014), but A-C-T was not (p = 0.1172). This suggests that LD may play a role in the association with *NSF *and *WNT3*. However, haplotype A-C-T frequency was higher in cases than in controls. While haplotype A-C-T association was not statistically significant, this association does support the possibility that a variant(s) in the *NSF *and *WNT3 *may contribute to PD, independent of *MAPT*. This association was replicated in the NINDS dataset, but not in the CIDR/PANKRATZ because the CIDR/PANKRATZ dataset lacked the SNP in *WNT3*. Taken together, there is suggestive evidence that *NSF *and *WNT3 *are candidate genes that need to be further studied.

**Table 3:** *MAPT-NSF *and *MAPT-WNT3 interaction *in the Ashkenazi Jewish Dataset: Multivariate logistic regression*

	*MAPT*	*NSF*	*WNT3*	Ashkenazi Jewish			NINDS			CIDR/Pankratz et al 2009		
Single point	rs1981997	rs183211	rs415430	P-value	OR	95% CI	P-value	OR	95% CI	P-value	OR	95% CI
	
	**A**			0.0009118	0.58	0.42-0.90	0.07504	0.86	0.73-1.01	0.0001569	0.72	0.61-0.86
		**T**		6.68 × 10^-5^	0.54	0.40-0.73	0.1015	0.87	0.74-1.02	0.001375	0.77	0.65-0.90
			**C**	8.59 × 10^-5^	0.54	0.30-0.74	0.002397	0.77	0.65-0.91		N/A	

2-mer	rs1981997	rs183211	rs415430	P-value	Freq. Cases	Freq. Controls	P-value	Freq. Cases	Freq. Controls	P-value	Freq. Cases	Freq. Controls
	
	**G**	**C**		**0.00034**	*0.7743*	0.6657	0.2634	*0.7654*	0.7491	**0.0003**	*0.791*	0.7386
	A	C		0.2118	*0.0187*	0.0084	0.1986	*0.0217*	0.0157	0.1059	0.0128	*0.0198*
	G	T		0.3211	0.0466	*0.0618*	0.2344	*0.0444*	0.0364	0.9238	0.0326	*0.0332*
	**A**	**T**		**0.00016**	0.1604	*0.264*	**0.0214**	0.1685	*0.1989*	**0.0007**	0.1636	*0.2084*
	
		**T**	**C**	**0.00023**	0.1559	*0.2558*	**0.0145**	0.1647	*0.1967*			
		**C**	**C**	0.2912	0.0456	*0.0617*	**0.0191**	0.0081	*0.0169*			
		T	T	0.2408	0.0512	*0.0701*	0.2133	*0.0482*	0.0395			
		**C**	**T**	**1.91 × 10^-5^**	*0.7473*	0.6125	**0.0269**	*0.7790*	0.7469		N/A	

3-mer	**A**	**T**	**C**	**0.00018**	0.156	*0.2585*	**0.0133**	0.1626	*0.1951*			
	**G**	**C**	**C**	0.3544	0.0445	*0.0584*	**0.0134**	0.0081	*0.0175*			
	G	T	T	0.2446	0.0450	*0.0628*	0.2758	*0.0422*	0.0350			
	A	C	T	0.1172	*0.0194*	0.0066	0.2187	*0.0222*	0.0163			
	**G**	**C**	**T**	**0.00014**	*0.7351*	0.6137	0.0518	*0.7649*	0.7361		N/A	

*Adjusted for age and sex. Age refers to age at onset for affected individuals, and age at examination for unaffected individuals. OR: odds ratios, 95% CI: 95% confidence interval; P-values for allelic/haplotype association; Single point shows the protective allele of each SNP; N/A, SNP rs415430 is not available in PD/CIDR data.

### LOC100505836 (3p24)

The SNP rs1694037, located in LOC100505836, was replicated in the CIDR dataset (p = 0.049) but not in the NINDS dataset (p = 0.849) and was not significant in the meta-analysis of all three datasets (Table 2).

### Intergenic SNP rs4745122 (Chr9q21.13)

The intergenic SNP, rs4745122, maps ~8.9kb proximal to *TMEM2*. This SNP was replicated in the NINDS (p = 0.007) but not the CIDR dataset (p = 0.748) and was significant in the meta analysis of all three datasets (p = 2.17 × 10^-4^).

### Replication of Previously identified PD Susceptibility Genes

In the analysis of the discovery AJ dataset, the previously identified PD susceptibility genes *MAPT, SNCA, LRRK2, GBA, PARK16, BST1, HLA, SYT11, ACMSD, STK39, LAMP3, GAK and CCDC6/HIP1R *were not included in the top 57 candidate SNPs/genes. *GBA *and *LRRK2 *were reported in the AJ sample derived from PD EPI study previously [22,30]. These genes also failed to reach genome wide significance in the NINDS and CIDR/Pankratz et al 2009 datasets. As shown in previous studies, it is not unexpected to miss risk genes in GWAS when SNP coverage is sparse in the candidate gene regions. Thus we further assessed association of SNPs at the chromosomal regions harbouring these genes in the AJ dataset and performed a meta-analysis including the NINDS and CIDR/Pankratz et al 2009 datasets. PD susceptibility genes that were associated in the AJ dataset are reported below.

#### *MAPT *(17q21.31)

We assessed associated SNPs and the presence of chromosome 17q21.31 alleles in the H1-H2 haplotype clades in *MAPT *using 'rs1981997' (HAPMAP CEU: 'A' = 0.208 and 'G' = 0.792) as a haplotype Tag SNP because the major (G) allele and the minor (A) allele of this SNP are fixed in the H1 and H2 clades respectively [31]. H1-H2 haplotype Tag SNP rs1981997 was associated with PD in the allelic and haplotype association analyses in both AJ and CIDR/Pankratz et al 2009 datasets. As discussed above, the present study supports the notion that in addition to *MAPT, NSF, WNT3*, or both contribute to PD susceptibility (Table 3).

#### *SNCA *(4q21)

In a recently published GWAS of PD in Caucasian subjects, *SNCA *showed the strongest association of 'top' SNPs analyzed (*SNCA *intron, rs356220, p = 2.7 × 10^-6^, OR = 1.48; 95% CI [1.25-1.74]) [15]. A meta-analysis of PD GWAS also confirmed the association (rs356219, p = 7.90 × 10^-26^). We assessed association of SNPs at the *SNCA *locus in all three datasets (Additional file 4). The SNP, rs11931074 (meta-analysis *p *value = 5.65 × 10^-5^), which maps near to *SNCA *was the most strongly associated SNP in the meta-analysis (data not shown). The same SNP (rs11931074) was also the most significantly associated SNP (*p *= 7.35 × 10^-17^, OR = 1.37) in a GWAS of PD in a Japanese population.

#### LRRK2 (12q12)

We assessed association of SNPs at the 12q12 region harbouring the *LRRK2 *gene in each dataset. SNPs within or near to *LRRK2 *did not reach genome wide significance in any of the datasets and were not included in the top '57' SNPs in the AJ dataset. However, we genotyped all subjects in the AJ dataset for the *LRRK2 *'G2019S' mutation, and observed an association of haplotypes consistent with previously published studies [20,30]. Strongest association was observed for the haplotype rs1427271-rs10735934-rs34637584 'GTA' (p = 7.66 × 10^-5^) (Additional file 4 for single point analysis; haplotype results not shown).

#### GBA (1q21)

**Figure 5:**
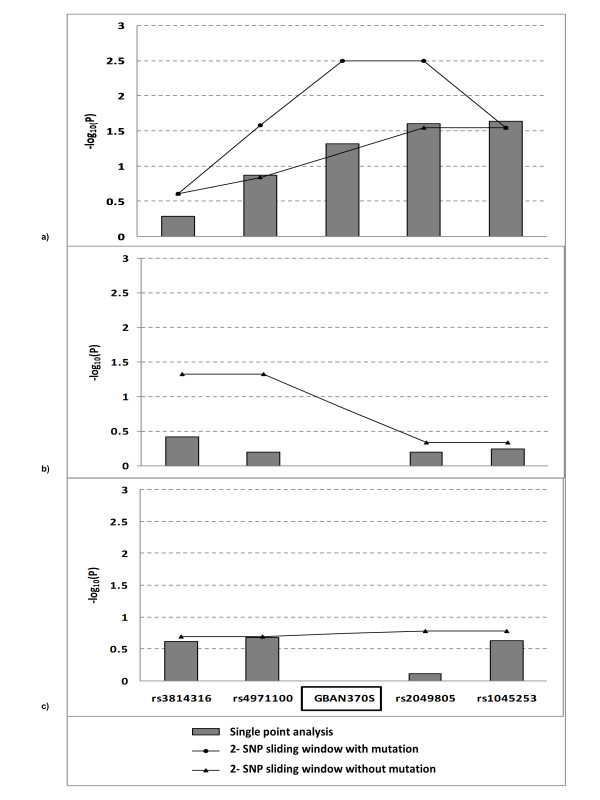
**Association and haplotype analysis of *GBA *mutation (*GBA *N370S) and flanking SNPs in 3 datasets**. a) Single point analysis plus 2 haplotype analyses: with vs. without the mutation in Ashkenazi Jewish. b) Single and haplotype analyses without mutation in NINDS. c) Single and haplotype analyses without mutation in CIDR/Pankratz et al 2009. 2-SNP sliding window, the strongest p-value was present from the exhaustive search.

Although SNPs within the *GBA *gene from the GWAS did not reach genome wide significance in any of the datasets analyzed we did observe strong association of SNPs and haplotypes at the *GBA *locus in our AJ dataset. When we assessed association of 8 SNPs at Chromosome 1q21 spanning a 74.4kb region from *TRIM46 *(rs4971100) to *SCAMP3 *(rs3180018) we observed that SNPs located in *GBA *were significantly associated with disease (i.e. rs2990245: OR = 1.39; p = 0.015) (Additional file 4). Using the SNPs from GWAS along with the *GBA *N370S allele (which was genotyped in all subjects), our 2-mer and 3-mer sliding window haplotype analyses flanking the *GBA *N370S allele revealed that a risk haplotype spanning ~12.5Kb of 'ATG' (*GBA *'N370S', rs2049805 and rs1045253) was associated with PD in the AJ dataset (p = 8.19 × 10^-4^) but not in the replication datasets (Figure 5). We previously reported that the GBA 'N370S' mutation is associated with PD [22].

#### *PARK16 *(1q32.1)

**Figure 6:**
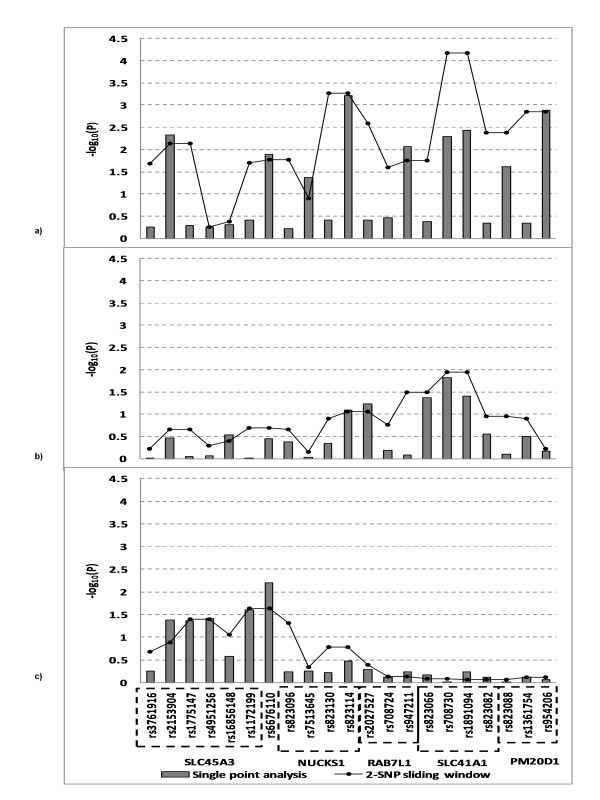
**Single point and haplotype analysis of *PARK16 *in Ashkenazi Jewish a), NINDS b) and CIDR/Pankratz et al 2009 c) datasets**. 2-SNP sliding window, the strongest p-value was present from the exhaustive search.

The *PARK16 *locus was previously identified as a susceptibility locus in a GWAS of PD in a Japanese population (*p *= 1.52 × 10^-12^) [13] and was also confirmed in a meta-analysis of PD GWAS. This region encompasses multiple genes; therefore, we assessed SNPs for association for a region spanning ~170 Kb (203905087-204074636 bp) at the *PARK16 *locus in all three datasets (Figure 6). In the AJ dataset the most strongly associated SNP, rs823114 (p = 6.12 × 10^-4^) was located in an intergenic region proximal to *NUCKS1*. Our analysis confirmed the finding of Satake et al (2009) [13] in a Japanese population in which they found that rs823114 (p = 2.7 × 10^-34) ^was strongly associated with transcript levels of *NUCKS1 *suggesting that this gene is a promising candidate for *PARK16*. However this SNP was not associated with PD in the replication datasets. In addition, two SNPs (rs708730 and rs1891094) in *SLC41A1 *were modestly associated with PD in the AJ dataset and NINDS dataset.

#### *BST1 *(4p15.32)

The *BST1 *gene was previously identified as a susceptibility gene in a GWAS of PD in a Japanese population [13] and was also confirmed in a meta-analysis of PD GWAS. On 4p15.32, four SNPs (rs11931532, rs12645693, rs4698412 and rs4538475) reached p < 5 × 10^-7 ^in the combined analysis (data not shown). These four SNPs showed strong disease association with almost the same significance levels (ranging from p = 3.94 × 10^-9 ^to p = 1.78 × 10^-8^, all OR = 1.24); among them, rs4538475 was the most strongly associated. The four SNPs were located from intron 8 to 4.1 kb downstream of *BST1 *(bone marrow stromal cell antigen). LD analysis revealed that the four SNPs were correlated with *r*^2 ^> 0.78 and lie within a 15 kb LD block containing *BST1* [13]. We assessed association of the four *BST1 *SNPs reported by Satake et al (2009) for association in all three datasets but none were significant. However the intronic *BST1 *SNP, rs12502586, was marginally associated with PD in the AJ (p = 0.027) and NINDS datasets (p = 0.014) and rs3213710 was strongly associated with PD in CIDR/Pankratz et al 2009 dataset (p = 6.39 × 10^-4^) (Additional file 4). Haplotype analysis in all three datasets confirmed the association with *BST1*.

#### *HLA (6p21.3)*

The HLA region was recently identified as a susceptibility locus in a late-onset sporadic PD population from North America [16]. We did not find evidence for association of SNPs at the *HLA-DRA *region with PD in AJ dataset (Additional file 4).

#### *STK39 *(Chr2q23.4)

The SNP rs2102808, located in the candidate gene *STK39 *was significant (p = 3.31 × 10^-11^) in a meta-analysis of PD GWAS. We assessed association of SNPs at the *STK39 *locus in the AJ dataset (Additional file 5). Two intronic SNPs, rs3754775 and rs6740826, located ~11 kb apart showed the strongest evidence of association in the AJ dataset (p = 0.005, OR = 2.12, 95% CI:1.24-3.62).

#### MCCC1/LAMP3

The SNP, rs1171141, located in the gene region *MCCC1/LAMP3 *was significant (p = 2.10 × 10^-8^) in a meta-analysis of PD GWAS. We assessed the association of SNPs at *MCCC1 *and *LAMP3*. The SNP, rs12493050, located in *LAMP3*, showed the strongest evidence of association in the AJ dataset (p = 0.005, OR = 0.64, CI: 0.47-0.88) (Additional file 5).

## Conclusions

This study identifies the candidate gene regions *LOC100505836, SLC25A48, UNC13B, SLCO3A1, WNT3*, and *NSF *as new candidates for PD using an AJ case-control population as a discovery dataset and two other large publicly available dataset as replication datasets. By utilizing a relatively genetically homogeneous AJ population and searching for variants that are rare (defined as a MAF threshold of 2% or higher), we report additional susceptibility variants for PD. In addition, we examined the magnitude of association of previously reported PD candidate genes including *MAPT, SNCA, LRRK2, GBA, PARK16, BST1, HLA, SYT11, ACMSD, STK39, MCCC1/LAMP3, GAK *and *CCDC62/HIP1R *in the AJ dataset and found them to be comparable to several reports in North American and European populations.

Of the new candidate genes that we identified in this study, many represent interesting candidates for PD based on function, as discussed below, and warrant additional follow up in independent studies and different PD populations. Functional studies suggest a role for three of the genes that we identified (*SLC25A48, UNC13B*, and *NSF*) in neuronal signalling and the dopamine pathway. *SLC25A48 *is a member of the solute carrier family 25 proteins that function as transporters of a large variety of molecules including ATP/ADP and amino acids [32]. Characterized SLC25s localize to the inner mitochondrial membrane and are also often referred to as mitochondrial carriers or uncoupling proteins (UCPs) [33]. *SLC25A48 *is highly expressed in the central nervous system (CNS) including the hypothalamus, pituitary and brainstem and has been shown to be important in healthy neurons for energy production and to have a role in neuronal signalling [34]. Previous studies have suggested a role for mitochondrial UCPs in PD, Alzheimer disease and amyotrophic lateral sclerosis [32].

The SNP rs10121009, located in *UNC13B *(*MUNC13*) was included in the top 57 SNPs in the AJ dataset and also showed evidence of strong association in a meta-analysis for all three datasets (p = 2.75 × 10^-6^). Experiments in C. elegans and mammalian cellular models systems suggest a role for the MUNC13 family of proteins in the priming of synaptic and secretory vesicles in a step just preceding fusion with the plasma membrane. *MUNC13 *has been shown to control the release of both neurotransmitters and neuropeptides from motorneurons in the Caenorhabditis elegans (C.elegans) neuromuscular junction [35]. The lipids and proteins involved in these networks are highly conserved between C. elegans and mammals.

Because *NSF *and *WNT3 *are closely located to *MAPT *and multiple datasets show support for possible association with PD, we examined this region encompassing *MAPT-NSF-WNT3 *further. Our data support the possibility that a variant(s) in the *NSF *and *WNT3 *may contribute to PD, independent of *MAPT*. This association was replicated in the NINDS dataset, but not in the CIDR/pankratz et al 2009 dataset because the CIDR/Pankratz et al 2009 dataset lacked the associated SNP in *WNT3*. Taken together and based on the function of these genes, there is suggestive evidence that *NSF *and *WNT3 *are candidate genes that need to be further studied. The function of *NSF *in vesicular trafficking and membrane fusion is well documented and the protein has also been shown to play a role in the fusion of synaptic vesicles in the presynaptic membrane during neurotransmission and to interact with neurotransmission receptors at the postsynaptic side [36]. More recent studies suggest an interaction between *NSF *and the Dopamine D1 receptor (D1R) which is important for the membrane localization of D1R [37]. D1R plays important roles in regulating motor coordination, working memory, learning and reward and D1R dysfunction is as associated with both psychiatric and neurological disorders including PD [38]. *WNT3 *is a member of the *WNT *gene family which encode secreted signaling proteins that play a role in several developmental processes, including embryonic and adult neurogenesis. Postnatal neurogenesis has been observed in two brain regions: the subventricular zone (SVZ) of the lateral ventricle and the subgranular zone (SGZ) of the dentate gyrus in the hippocampus, among vertebrates including human. Genetic factors essential for neural development including WNT3 are also expressed in adult neurogenic regions. Cell proliferation of neural progenitors in the SVZ of PD patients and animal models has been shown to be decreased and modulated by dopamine.

We also replicate association of several previously identified PD genes and loci in our AJ population including *MAPT, SNCA, LRRK2, GBA, PARK16, BST1, STK39 *and *LAMP3*. Both *LRRK2 *and *GBA *represent the most common risk factors in the AJ PD population. In the AJ dataset, we observed a significant association for the *LRRK2 *'G2019S' mutation as well as for a single haplotype, and these findings are consistent with previously published studies. Among 268 PD cases, 31 (11.6%) individuals carried the *LRRK2 G2019S *mutation, and their mean age at onset was younger/similar to non-carriers (mean age at onset of 56.5 (SD = 11.1) vs. 60.3 (SD = 12.3), respectively). The *GBA *'N370S' mutation is the most common allele reported in AJ PD cases in several studies however a risk haplotype supporting a founder effect has not been previously reported. In our AJ PD dataset we identified a risk haplotype of 'ATG' (*GBA *'N370S', rs2049805 and rs1045253)(p = 8.19 × 10^-4^) spanning ~12.5 Kb suggesting that these individuals share a common founder. Among 268 PD cases, 28 individuals carried the *GBA N370S *mutation (10.4%), and their mean age at onset was younger/similar to non-carriers (mean age at onset of 57.4 (SD = 12.4) vs. 60.2 (SD = 12.1), respectively).

Our analysis of the *PARK16 *locus in our AJ dataset confirms the finding of Satake et al (2009) [13] in a Japanese population and suggests that *NUCKS1 *is a promising candidate for *PARK16*. More recently, Tucci et al (2010) [39] analysed the coding regions of 3 candidate genes (*NUCKS1, RAB7L1*, and *SLC41A1*) at *PARK16 *in a British cohort of 182 PD patients. Novel mutations were identified in 1 PD patient in *RAB7L1 *(K157R) and in another patient in *SLC41A1 *(A350 V). Follow-up studies including re-sequencing of the *NUCKS1 *gene and other candidate genes at the *PARK16 *region are warranted.

In summary, our GWAS study has identified candidate gene regions for PD that are implicated in neuronal signalling and the dopamine pathway. Although the power to detect genome-wide level significance in the AJ dataset was low because of the small sample size we have demonstrated the utility of this dataset in gene and SNP discovery both by replication in dbGaP datasets with a larger sample size combined with joint analyses and by replicating association of previously identified PD susceptibility genes. Follow-up genotyping, replication studies and sequencing will be needed to confirm our findings in future studies.

## Competing interests

The authors declare that they have no competing interests.

## Authors' contributions

LNC and JH designed the study. EL, LC, CW, BF, SF and KM contributed samples. Statistical analysis was performed by XL, RC and JH. LC, XL, RC, JH, MV, SK, HA, and HM participated in the analysis. LC, JL, RC and XL wrote the paper. All co-authors contributed to the preparation of the paper. All authors read and approved the final manuscript.

## Pre-publication history

The pre-publication history for this paper can be accessed here:

http://www.biomedcentral.com/1471-2350/12/104/prepub

## Supplementary Material

Additional file 1 **'Top' 57 SNPs with P value < 9.9 × 10^-5 ^identified in the Ashkenazi Jewish PD GWAS**. 'Top' 57 SNPs with P value < 9.9 × 10^-5 ^identified in the Ashkenazi Jewish PD GWAS. OR: odds ratios, 95% CI: 95% confidence interval. Click here for file

Additional file 2 **SNPs reaching Genome Wide Significance in the NINDS and CIDR/Pankratz et al 2009 datasets**. SNPs reaching Genome Wide Significance in the NINDS and CIDR/Pankratz et al 2009 datasets. OR: odds ratios, 95% CI: 95% confidence interval. Click here for file

Additional file 3 **Haplotype analysis of *LOC15328/SLC25A48, UNC13B, SLCO3A1, WNT3 *in 2 or 3 datasets**. **a). Haplotype analysis of *LOC15328/SLC25A48 *in 3 datasets. These haplotype results **in Additional file 3 a), b), c) and d) are further evaluation of the SNPs in Table 2. *SNP 1, rs2304075; 2, rs6596270; 3, rs4976493; 4, rs4246802; 5, rs7717673. **b) Haplotype analysis of *UNC13B *in 3 datasets**. *SNP 1, rs7036061; 2, rs7040048; 3, rs10121009; 4, rs10114937; 5, rs10758303. **c) Haplotype analysis of *SLCO3A1 *in 3 datasets**. *SNP 1, rs1400784; 2, rs2387400; 3, rs7171137; 4, rs12913189; 5, rs6496888. **d) Haplotype analysis of *WNT3 *in Ashkenazi Jewish and NINDS**. *SNP 1, rs12325819; 2, rs199533; 3, rs415430; 4, rs2074404; 5, rs199494; rs415430 is not on the CIDR/Pankratz et al 2009 dataset. Click here for file

Additional file 4 **Single SNP association of the genes reported in other studies: *GBA, BST1, SNCA, HLA-DRA and LRRK2***. Single SNP association of the genes reported in other studies: *GBA, BST1, SNCA, HLA-DRA and LRRK2*. F_A: Minor allele freq. of case, F_U: Minor allele freq. of control, OR: odds ratios, 95% CI: 95% confidence interval. Click here for file

Additional file 5 **Single SNP association of the genes reported by the International Parkinson Disease Genomics Consortium: *STK39, MCCC1 and LAMP3***. Single SNP association of the genes reported by the International Parkinson Disease Genomics Consortium: *STK39, MCCC1 and LAMP3*. OR: odds ratios, 95% CI: 95% confidence interval Click here for file

## References

[B1] Polymeropoulos MH Lavedan C Leroy E Ide SE Dehejia A Dutra A Pike B Root H Rubenstein J Boyer R Stenroos ES Chandrasekharappa S Athanassiadou A Papapetropoulos T Johnson WG Lazzarini AM Duvoisin RC Di Iorio G Golbe LI Nussbaum RL Mutation in the alpha-synuclein gene identified in families with Parkinson's disease Science 1997 276 5321 2045 2047 10.1126/science.276.5321.2045 9197268

[B2] Kitada T Asakawa S Hattori N Matsumine H Yamamura Y Minoshima S Yokochi M Mizuno Y Shimizu N Mutations in the parkin gene cause autosomal recessive juvenile parkinsonism Nature 1998 392 6676 605 608 10.1038/33416 9560156

[B3] Valente EM Abou-Sleiman PM Caputo V Muqit MM Harvey K Gispert S Ali Z Del Turco D Bentivoglio AR Healy DG Albanese A Nussbaum R Gonzalez-Maldonado R Deller T Salvi S Cortelli P Gilks WP Latchman DS Harvey RJ Dallapiccola B Auburger G Wood NW Hereditary early-onset Parkinson's disease caused by mutations in PINK1 Science 2004 304 5674 1158 1160 10.1126/science.1096284 15087508

[B4] Bonifati V Rizzu P van Baren MJ Schaap O Breedveld GJ Krieger E Dekker MC Squitieri F Ibanez P Joosse M van Dongen JW Vanacore N van Swieten JC Brice A Meco G van Duijn CM Oostra BA Heutink P Mutations in the DJ-1 gene associated with autosomal recessive early-onset parkinsonism Science 2003 299 5604 256 259 10.1126/science.1077209 12446870

[B5] Paisan-Ruiz C Jain S Evans EW Gilks WP Simon J van der Brug M Lopez de Munain A Aparicio S Gil AM Khan N Johnson J Martinez JR Nicholl D Carrera IM Pena AS de Silva R Lees A Marti-Masso JF Perez-Tur J Wood NW Singleton AB Cloning of the gene containing mutations that cause PARK8-linked Parkinson's disease Neuron 2004 44 4 595 600 10.1016/j.neuron.2004.10.023 15541308

[B6] Lees AJ Hardy J Revesz T Parkinson's disease Lancet 2009 373 9680 2055 2066 10.1016/S0140-6736(09)60492-X 19524782

[B7] Nuytemans K Theuns J Cruts M Van Broeckhoven C Genetic etiology of Parkinson disease associated with mutations in the SNCA, PARK2, PINK1, PARK7, and LRRK2 genes: a mutation update Hum Mutat 2010 31 7 763 780 10.1002/humu.21277 20506312 PMC3056147

[B8] Saad M Lesage S Saint-Pierre A Corvol JC Zelenika D Lambert JC Vidailhet M Mellick GD Lohmann E Durif F Pollak P Damier P Tison F Silburn PA Tzourio C Forlani S Loriot MA Giroud M Helmer C Portet F Amouyel P Lathrop M Elbaz A Durr A Martinez M Brice A Genome-wide association study confirms BST1 and suggests a locus on 12q24 as the risk loci for Parkinson's disease in the European population Hum Mol Genet 2011 20 3 615 627 10.1093/hmg/ddq497 21084426

[B9] Spencer CC Plagnol V Strange A Gardner M Paisan-Ruiz C Band G Barker RA Bellenguez C Bhatia K Blackburn H Blackwell JM Bramon E Brown MA Burn D Casas JP Chinnery PF Clarke CE Corvin A Craddock N Deloukas P Edkins S Evans J Freeman C Gray E Hardy J Hudson G Hunt S Jankowski J Langford C Lees AJ Dissection of the genetics of Parkinson's disease identifies an additional association 5' of SNCA and multiple associated haplotypes at 17q21 Hum Mol Genet 2011 20 2 345 353 21044948 10.1093/hmg/ddq469 PMC3005904

[B10] Maraganore DM de Andrade M Lesnick TG Strain KJ Farrer MJ Rocca WA Pant PV Frazer KA Cox DR Ballinger DG High-resolution whole-genome association study of Parkinson disease Am J Hum Genet 2005 77 5 685 693 10.1086/496902 16252231 PMC1271381

[B11] Fung HC Scholz S Matarin M Simon-Sanchez J Hernandez D Britton A Gibbs JR Langefeld C Stiegert ML Schymick J Okun MS Mandel RJ Fernandez HH Foote KD Rodriguez RL Peckham E De Vrieze FW Gwinn-Hardy K Hardy JA Singleton A Genome-wide genotyping in Parkinson's disease and neurologically normal controls: first stage analysis and public release of data Lancet Neurol 2006 5 11 911 916 10.1016/S1474-4422(06)70578-6 17052657

[B12] Latourelle JC Pankratz N Dumitriu A Wilk JB Goldwurm S Pezzoli G Mariani CB DeStefano AL Halter C Gusella JF Nichols WC Myers RH Foroud T Genomewide association study for onset age in Parkinson disease BMC Med Genet 2009 10 98 10.1186/1471-2350-10-98 19772629 PMC2758866

[B13] Satake W Nakabayashi Y Mizuta I Hirota Y Ito C Kubo M Kawaguchi T Tsunoda T Watanabe M Takeda A Tomiyama H Nakashima K Hasegawa K Obata F Yoshikawa T Kawakami H Sakoda S Yamamoto M Hattori N Murata M Nakamura Y Toda T Genome-wide association study identifies common variants at four loci as genetic risk factors for Parkinson's disease Nat Genet 2009 41 12 1303 1307 10.1038/ng.485 19915576

[B14] Simon-Sanchez J Schulte C Bras JM Sharma M Gibbs JR Berg D Paisan-Ruiz C Lichtner P Scholz SW Hernandez DG Kruger R Federoff M Klein C Goate A Perlmutter J Bonin M Nalls MA Illig T Gieger C Houlden H Steffens M Okun MS Racette BA Cookson MR Foote KD Fernandez HH Traynor BJ Schreiber S Arepalli S Zonozi R Genome-wide association study reveals genetic risk underlying Parkinson's disease Nat Genet 2009 41 12 1308 1312 10.1038/ng.487 19915575 PMC2787725

[B15] Edwards TL Scott WK Almonte C Burt A Powell EH Beecham GW Wang L Zuchner S Konidari I Wang G Singer C Nahab F Scott B Stajich JM Pericak-Vance M Haines J Vance JM Martin ER Genome-wide association study confirms SNPs in SNCA and the MAPT region as common risk factors for Parkinson disease Ann Hum Genet 2010 74 2 97 109 10.1111/j.1469-1809.2009.00560.x 20070850 PMC2853717

[B16] Hamza TH Zabetian CP Tenesa A Laederach A Montimurro J Yearout D Kay DM Doheny KF Paschall J Pugh E Kusel VI Collura R Roberts J Griffith A Samii A Scott WK Nutt J Factor SA Payami H Common genetic variation in the HLA region is associated with late-onset sporadic Parkinson's disease Nat Genet 2010 42 9 781 785 10.1038/ng.642 20711177 PMC2930111

[B17] Consortium IPDG Imputation of sequence variants for identification of genetic risks for Parkinson's disease: a meta-analysis of genome-wide association studies The Lancet 2011 377 9766 641 649 10.1016/S0140-6736(10)62345-8 PMC3696507 21292315

[B18] Ostrer H A genetic profile of contemporary Jewish populations Nat Rev Genet 2001 2 11 891 898 10.1038/35098506 11715044

[B19] Atzmon G Hao L Pe'er I Velez C Pearlman A Palamara PF Morrow B Friedman E Oddoux C Burns E Ostrer H Abraham's children in the genome era: major Jewish diaspora populations comprise distinct genetic clusters with shared Middle Eastern Ancestry Am J Hum Genet 2010 86 6 850 859 10.1016/j.ajhg.2010.04.015 20560205 PMC3032072

[B20] Ozelius LJ Senthil G Saunders-Pullman R Ohmann E Deligtisch A Tagliati M Hunt AL Klein C Henick B Hailpern SM Lipton RB Soto-Valencia J Risch N Bressman SB LRRK2 G2019S as a cause of Parkinson's disease in Ashkenazi Jews N Engl J Med 2006 354 4 424 425 10.1056/NEJMc055509 16436782

[B21] Bar-Shira A Hutter CM Giladi N Zabetian CP Orr-Urtreger A Ashkenazi Parkinson's disease patients with the LRRK2 G2019S mutation share a common founder dating from the second to fifth centuries Neurogenetics 2009 10 4 355 358 10.1007/s10048-009-0186-0 19283415

[B22] Clark LN Ross BM Wang Y Mejia-Santana H Harris J Louis ED Cote LJ Andrews H Fahn S Waters C Ford B Frucht S Ottman R Marder K Mutations in the glucocerebrosidase gene are associated with early-onset Parkinson disease Neurology 2007 69 12 1270 1277 10.1212/01.wnl.0000276989.17578.02 17875915 PMC3624967

[B23] Sidransky E Nalls MA Aasly JO Aharon-Peretz J Annesi G Barbosa ER Bar-Shira A Berg D Bras J Brice A Chen CM Clark LN Condroyer C De Marco EV Durr A Eblan MJ Fahn S Farrer MJ Fung HC Gan-Or Z Gasser T Gershoni-Baruch R Giladi N Griffith A Gurevich T Januario C Kropp P Lang AE Lee-Chen GJ Lesage S Multicenter analysis of glucocerebrosidase mutations in Parkinson's disease N Engl J Med 2009 361 17 1651 1661 10.1056/NEJMoa0901281 19846850 PMC2856322

[B24] Pankratz N Wilk JB Latourelle JC DeStefano AL Halter C Pugh EW Doheny KF Gusella JF Nichols WC Foroud T Myers RH Genomewide association study for susceptibility genes contributing to familial Parkinson disease Hum Genet 2009 124 6 593 605 10.1007/s00439-008-0582-9 18985386 PMC2627511

[B25] Marder K Levy G Louis ED Mejia-Santana H Cote L Andrews H Harris J Waters C Ford B Frucht S Fahn S Ottman R Familial aggregation of early- and late-onset Parkinson's disease Ann Neurol 2003 54 4 507 513 10.1002/ana.10711 14520664

[B26] Purcell S Neale B Todd-Brown K Thomas L Ferreira MA Bender D Maller J Sklar P de Bakker PI Daly MJ Sham PC PLINK: a tool set for whole-genome association and population-based linkage analyses Am J Hum Genet 2007 81 3 559 575 10.1086/519795 17701901 PMC1950838

[B27] Hartl DL Clark AG Principles of population genetics 2007 4 Sunderland, Mass.: Sinauer Associates

[B28] Ge D Zhang K Need AC Martin O Fellay J Urban TJ Telenti A Goldstein DB WGAViewer: software for genomic annotation of whole genome association studies Genome Res 2008 18 4 640 643 10.1101/gr.071571.107 18256235 PMC2279251

[B29] Johnson AD Handsaker RE Pulit SL Nizzari MM O'Donnell CJ de Bakker PI SNAP: a web-based tool for identification and annotation of proxy SNPs using HapMap Bioinformatics 2008 24 24 2938 2939 10.1093/bioinformatics/btn564 18974171 PMC2720775

[B30] Clark LN Wang Y Karlins E Saito L Mejia-Santana H Harris J Louis ED Cote LJ Andrews H Fahn S Waters C Ford B Frucht S Ottman R Marder K Frequency of LRRK2 mutations in early- and late-onset Parkinson disease Neurology 2006 67 10 1786 1791 10.1212/01.wnl.0000244345.49809.36 17050822

[B31] Stefansson H Helgason A Thorleifsson G Steinthorsdottir V Masson G Barnard J Baker A Jonasdottir A Ingason A Gudnadottir VG Desnica N Hicks A Gylfason A Gudbjartsson DF Jonsdottir GM Sainz J Agnarsson K Birgisdottir B Ghosh S Olafsdottir A Cazier JB Kristjansson K Frigge ML Thorgeirsson TE Gulcher JR Kong A Stefansson K A common inversion under selection in Europeans Nat Genet 2005 37 2 129 137 10.1038/ng1508 15654335

[B32] Haitina T Lindblom J Renstrom T Fredriksson R Fourteen novel human members of mitochondrial solute carrier family 25 (SLC25) widely expressed in the central nervous system Genomics 2006 88 6 779 790 10.1016/j.ygeno.2006.06.016 16949250

[B33] Palmieri F The mitochondrial transporter family (SLC25): physiological and pathological implications Pflugers Arch 2004 447 5 689 709 10.1007/s00424-003-1099-7 14598172

[B34] Richard D Clavel S Huang Q Sanchis D Ricquier D Uncoupling protein 2 in the brain: distribution and function Biochem Soc Trans 2001 29 (Pt 6):812 817 10.1042/0300-5127:0290812 11709080

[B35] Perez-Mansilla B Nurrish S A network of G-protein signaling pathways control neuronal activity in C. elegans Adv Genet 2009 65 145 192 19615533 10.1016/S0065-2660(09)65004-5

[B36] Haas A NSF--fusion and beyond Trends Cell Biol 1998 8 12 471 473 10.1016/S0962-8924(98)01388-9 9861668

[B37] Chen S Liu F Interaction of dopamine D1 receptor with N-ethylmaleimide-sensitive factor is important for the membrane localization of the receptor J Neurosci Res 2010 88 11 2504 2512 20623535 10.1002/jnr.22401

[B38] Fiorentini C Busi C Spano P Missale C Dimerization of dopamine D1 and D3 receptors in the regulation of striatal function Curr Opin Pharmacol 2010 10 1 87 92 10.1016/j.coph.2009.09.008 19837631

[B39] Tucci A Nalls MA Houlden H Revesz T Singleton AB Wood NW Hardy J Paisan-Ruiz C Genetic variability at the PARK16 locus Eur J Hum Genet 2010 18 12 1356 1359 10.1038/ejhg.2010.125 20683486 PMC3002857

