# Supplementary material for: Genome-Wide association study identifies candidate genes for Parkinson's disease in an Ashkenazi Jewish population
Source: BMC Med Genet. 2011 Aug 3;12:104. doi: 10.1186/1471-2350-12-104 (PMC3166909; doi:10.1186/1471-2350-12-104)
Supplement: Additional file 1 — 'Top' 57 SNPs with P value < 9.9 × 10^-5^ identified in the Ashkenazi Jewish PD GWAS. 'Top' 57 SNPs with P value < 9.9 × 10^-5^ identified in the Ashkenazi Jewish PD GWAS. OR: odds ratios, 95% CI: 95% confidence interval. [file 1471-2350-12-104-S1.DOC]

**Additional file 1**

| **CHR** | **SNP** | **BP** | **Minor/Major Allele** | **Freq. Case** | **Freq. Control** | **P** | **OR** | **95% CI** | **Gene** |
| --- | --- | --- | --- | --- | --- | --- | --- | --- | --- |
| 1 | rs10921184 | 187295821 | C/T | 0.496 | 0.362 | 7.88×10^-5^ | 1.74 | 1.32-2.29 | Intergenic |
| 1 | rs12734001 | 200657537 | A/G | 0.326 | 0.197 | 4.03×10^-5^ | 1.97 | 1.42-2.72 | *PPP1R12B* |
| 2 | rs12469652 | 36830620 | C/T | 0.291 | 0.424 | 3.95×10^-5^ | 0.56 | 0.42-0.74 | *VIT* |
| 2 | rs12613026 | 42867793 | G/A | 0.468 | 0.316 | 6.49×10^-6^ | 1.91 | 1.44-2.52 | *HAAO* |
| 2 | rs13003114 | 180085724 | G/A | 0.25 | 0.371 | 8.92×10^-5^ | 0.56 | 0.42-0.75 | *ZNF385B* |
| 3 | rs1352135 | 21935471 | C/T | 0.097 | 0.202 | 8.78×10^-6^ | 0.42 | 0.29-0.62 | Intergenic |
| 3 | rs1684524 | 21936271 | T/G | 0.095 | 0.201 | 7.74×10^-6^ | 0.42 | 0.28-0.62 | Intergenic |
| 3 | rs1694037 | 21941114 | T/C | 0.096 | 0.199 | 1.02×10^-5^ | 0.42 | 0.29-0.62 | Intergenic |
| 3 | rs17583756 | 21979879 | A/C | 0.107 | 0.205 | 4.63×10^-5^ | 0.46 | 0.32-0.67 | Intergenic |
| 3 | rs9871168 | 21987289 | G/A | 0.108 | 0.205 | 5.92×10^-5^ | 0.47 | 0.32-0.68 | Intergenic |
| 3 | rs4678649 | 32992228 | T/C | 0.138 | 0.242 | 8.54×10^-5^ | 0.50 | 0.36-0.71 | Intergenic |
| 3 | rs1317243 | 113535936 | C/T | 0.478 | 0.346 | 9.21×10^-5^ | 1.73 | 1.31-2.28 | *CD200* |
| 3 | rs9867544 | 113561967 | C/T | 0.367 | 0.508 | 3.08×10^-5^ | 0.56 | 0.43-0.74 | *CD200* |
| 3 | rs7641311 | 113574386 | G/A | 0.360 | 0.511 | 8.17×10^-6^ | 0.54 | 0.41-0.71 | Intergenic |
| 3 | rs1879512 | 113576590 | G/A | 0.410 | 0.571 | 3.04×10^-6^ | 0.52 | 0.40-0.69 | Intergenic |
| 3 | rs11714053 | 133332100 | A/G | 0.147 | 0.253 | 7.98×10^-5^ | 0.51 | 0.36-0.71 | Intergenic |
| 4 | rs4496609 | 61324159 | A/G | 0.043 | 0.121 | 1.22×10^-5^ | 0.33 | 0.19-0.55 | Intergenic |
| 4 | rs7659598 | 182936373 | T/C | 0.015 | 0.068 | 3.65×10^-5^ | 0.21 | 0.09-0.47 | Intergenic |
| 5 | rs1916642 | 72488303 | T/C | 0.243 | 0.388 | 3.41×10^-6^ | 0.51 | 0.38-0.68 | Intergenic |
| 5 | rs6879012 | 72498637 | A/G | 0.250 | 0.390 | 7.78×10^-6^ | 0.52 | 0.39-0.69 | Intergenic |
| 5 | rs4976493 | 135210022 | A/G | 0.442 | 0.309 | 6.66×10^-5^ | 1.77 | 1.34-2.35 | *SLC25A48* |
| 6 | rs1548240 | 77877214 | A/G | 0.394 | 0.267 | 8.80×10^-5^ | 1.79 | 1.34-2.40 | Intergenic |
| 7 | rs2266920 | 2741704 | T/C | 0.114 | 0.211 | 5.76×10^-5^ | 0.47 | 0.32-0.68 | *GNA12* |
| 8 | rs3808386 | 97342719 | T/C | 0.396 | 0.264 | 5.37×10^-5^ | 1.82 | 1.36-2.44 | *MTERFD1* |
| 8 | rs7464066 | 144401827 | C/T | 0.276 | 0.416 | 1.35×10^-5^ | 0.53 | 0.40-0.71 | *ZFP41* |
| 8 | rs7815716 | 144408731 | G/T | 0.276 | 0.416 | 1.35×10^-5^ | 0.53 | 0.40-0.71 | *ZFP41* |
| 8 | rs10090179 | 144410428 | T/C | 0.277 | 0.416 | 1.60×10^-5^ | 0.54 | 0.40-0.71 | *ZFP41* |
| 9 | rs10121009 | 35259819 | A/G | 0.131 | 0.236 | 5.32×10^-5^ | 0.49 | 0.34-0.69 | *UNC13B* |
| 9 | rs4745122 | 73582507 | A/C | 0.351 | 0.225 | 5.48×10^-5^ | 1.87 | 1.38-2.54 | Intergenic |
| 10 | rs1194491 | 53636130 | G/T | 0.468 | 0.331 | 5.64×10^-5^ | 1.77 | 1.34-2.33 | *PRKG1* |
| 10 | rs10999501 | 72171365 | A/G | 0.188 | 0.081 | 9.75×10^-6^ | 2.61 | 1.69-4.04 | *ADAMTS14* |
| 10 | rs12221267 | 72175772 | T/C | 0.185 | 0.087 | 5.07×10^-5^ | 2.38 | 1.55-3.66 | *ADAMTS14* |
| 11 | rs7129006 | 22760288 | G/A | 0.366 | 0.503 | 4.61×10^-5^ | 0.57 | 0.43-0.75 | *GAS2* |
| 11 | rs1895824 | 43056523 | C/T | 0.254 | 0.393 | 1.05×10^-5^ | 0.52 | 0.39-0.70 | Intergenic |
| 11 | rs7935833 | 43057836 | C/T | 0.202 | 0.326 | 2.95×10^-5^ | 0.52 | 0.38-0.71 | Intergenic |
| 11 | rs1365084 | 43066630 | G/A | 0.254 | 0.393 | 1.05×10^-5^ | 0.52 | 0.39-0.70 | Intergenic |
| 11 | rs2186580 | 101030083 | A/C | 0.065 | 0.148 | 4.69×10^-5^ | 0.40 | 0.25-0.63 | Intergenic |
| 11 | rs10895174 | 101046434 | G/A | 0.063 | 0.141 | 9.72×10^-5^ | 0.41 | 0.26-0.65 | Intergenic |
| 12 | rs10881029 | 45729804 | G/A | 0.289 | 0.419 | 6.19×10^-5^ | 0.56 | 0.43-0.75 | Intergenic |
| 12 | rs2158133 | 45736697 | C/T | 0.289 | 0.419 | 6.19×10^-5^ | 0.56 | 0.43-0.75 | Intergenic |
| 12 | rs4913250 | 66214491 | A/G | 0.224 | 0.346 | 6.84×10^-5^ | 0.55 | 0.41-0.74 | Intergenic |
| 15 | rs8030609 | 31330560 | A/G | 0.295 | 0.169 | 1.78×10^-5^ | 2.06 | 1.48-2.88 | Intergenic |
| 15 | rs7171137 | 90363631 | A/C | 0.397 | 0.264 | 3.79×10^-5^ | 1.84 | 1.38-2.47 | *SLCO3A1* |
| 16 | rs1881335 | 5206420 | C/A | 0.123 | 0.239 | 5.99×10^-6^ | 0.45 | 0.31-0.64 | Intergenic |
| 16 | rs9925078 | 5209254 | G/T | 0.191 | 0.309 | 5.14×10^-5^ | 0.53 | 0.39-0.72 | Intergenic |
| 16 | rs7201500 | 6837660 | T/C | 0.500 | 0.367 | 9.69×10^-5^ | 1.72 | 1.31-2.27 | *A2BP1* |
| 17 | rs183211 | 42143493 | T/C | 0.207 | 0.326 | 7.16×10^-5^ | 0.54 | 0.40-0.73 | *NSF* |
| 17 | rs415430 | 42214305 | C/T | 0.202 | 0.317 | 8.45×10^-5^ | 0.54 | 0.40-0.74 | *WNT3* |
| 18 | rs11661054 | 72833263 | A/C | 0.436 | 0.587 | 1.01×10^-5^ | 0.54 | 0.41-0.71 | *MBP* |
| 19 | rs12985786 | 13328036 | T/C | 0.028 | 0.088 | 8.86×10^-5^ | 0.30 | 0.16-0.57 | *CACNA1A* |
| 19 | rs10415765 | 18309105 | C/T | 0.274 | 0.155 | 3.16×10^-5^ | 2.06 | 1.46-2.91 | Intergenic |
| 20 | rs151358 | 57043454 | A/C | 0.213 | 0.357 | 2.24×10^-6^ | 0.49 | 0.36-0.66 | *SLMO2* |
| 21 | rs225376 | 42547191 | G/C | 0.243 | 0.370 | 4.30×10^-5^ | 0.54 | 0.41-0.73 | *ABCG1* |
| 21 | rs7282991 | 45605451 | A/G | 0.076 | 0.164 | 5.43×10^-5^ | 0.42 | 0.28-0.65 | Intergenic |
| 21 | rs2183593 | 45616530 | A/G | 0.076 | 0.164 | 5.43×10^-5^ | 0.42 | 0.28-0.65 | Intergenic |
| X | rs2843518 | 123515881 | C/T | 0.199 | 0.339 | 5.63×10^-5^ | 0.48 | 0.34-0.69 | *ODZ1* |
| X | rs2843520 | 123516714 | C/T | 0.199 | 0.339 | 6.37×10^-5^ | 0.48 | 0.34-0.69 | *ODZ1* |
