# Supplementary material for: Genome-Wide association study identifies candidate genes for Parkinson's disease in an Ashkenazi Jewish population
Source: BMC Med Genet. 2011 Aug 3;12:104. doi: 10.1186/1471-2350-12-104 (PMC3166909; doi:10.1186/1471-2350-12-104)
Supplement: Additional file 2 — SNPs reaching Genome Wide Significance in the NINDS and CIDR/Pankratz et al 2009 datasets. SNPs reaching Genome Wide Significance in the NINDS and CIDR/Pankratz et al 2009 datasets. OR: odds ratios, 95% CI: 95% confidence interval. [file 1471-2350-12-104-S2.DOC]

**Additional file 2**

| **CHR** | **SNP** | **BP** | **Minor/Major Allele** | **Freq. Case** | **Freq. Control** | **P** | **OR** | **95% CI** | **Gene** | **Database** |
| --- | --- | --- | --- | --- | --- | --- | --- | --- | --- | --- |
| 1 | rs11209290 | 68643615 | A/G | 0.065 | 0.021 | 8.87×10^-10^ | 3.27 | 2.19-4.87 | Intergenic |  |
| 8 | rs16938508 | 74013366 | G/A | 0.074 | 0.029 | 2.31×10^-8^ | 2.62 | 1.85-3.72 | Intergenic | NINDS |
| 16 | rs3784847 | 60534950 | C/T | 0.104 | 0.051 | 1.81×10^-8^ | 2.18 | 1.65-2.87 | *CDH8* |  |
| 18 | rs8086137 | 26961413 | C/T | 0.083 | 0.028 | 2.35×10^-11^ | 3.09 | 2.18-4.36 | Intergenic |  |
| 13 | rs2451078 | 18996289 | C/G | 0.318 | 0.219 | 1.94×10^-10^ | 1.66 | 1.42-1.94 | *TPTE2* | CIDR/Pankratz et al 2009 |
