# Supplementary material for: Genome-Wide association study identifies candidate genes for Parkinson's disease in an Ashkenazi Jewish population
Source: BMC Med Genet. 2011 Aug 3;12:104. doi: 10.1186/1471-2350-12-104 (PMC3166909; doi:10.1186/1471-2350-12-104)
Supplement: Additional file 3 — Haplotype analysis of LOC15328/SLC25A48, UNC13B, SLCO3A1, WNT3 in 2 or 3 datasets. a). Haplotype analysis of LOC15328/SLC25A48 in 3 datasets. These haplotype results in Additional file 3 a), b), c) and d) are further evaluation of the SNPs in Table 2. *SNP 1, rs2304075; 2, rs6596270; 3, rs4976493; 4, rs4246802; 5, rs7717673. b) Haplotype analysis of UNC13B in 3 datasets. *SNP 1, rs7036061; 2, rs7040048; 3, rs10121009; 4, rs10114937; 5, rs10758303. c) Haplotype analysis of SLCO3A1 in 3 datasets. *SNP 1, rs1400784; 2, rs2387400; 3, rs7171137; 4, rs12913189; 5, rs6496888. d) Haplotype analysis of WNT3 in Ashkenazi Jewish and NINDS. *SNP 1, rs12325819; 2, rs199533; 3, rs415430; 4, rs2074404; 5, rs199494; rs415430 is not on the CIDR/Pankratz et al 2009 dataset. [file 1471-2350-12-104-S3.DOC]

**Additional file 3a**

| SNP* | | | | | **Ashkenazi Jewish** | | | | | **NINDS** | | | | | **CIDR/Pankratz et al 2009** | | | | |
| --- | --- | --- | --- | --- | --- | --- | --- | --- | --- | --- | --- | --- | --- | --- | --- | --- | --- | --- | --- |
| 1 | 2 | 3 | 4 | 5 | **Freq. Case** | **Freq. Control** | **χ^2^** | **P** | **Global**  **P** | **Freq. Case** | **Freq. Control** | **χ^2^** | **P** | **Global P** | **Freq. Case** | **Freq. Control** | **χ^2^** | **P** | **Global P** |
| C | C |  |  |  | 0.246 | 0.232 | 0.25 | 0.617 | 0.004 | 0.210 | 0.212 | 0.02 | 0.877 | 0.027 | 0.209 | 0.226 | 1.40 | 0.237 | 0.425 |
| **T** | **T** |  |  |  | **0.226** | **0.144** | **9.12** | **0.003** |  | **0.207** | **0.171** | **6.93** | **0.008** |  | 0.173 | 0.176 | 0.06 | 0.799 |  |
| C | T |  |  |  | 0.528 | 0.624 | 8.05 | 0.005 |  | 0.583 | 0.617 | 3.92 | 0.048 |  | 0.618 | 0.599 | 1.43 | 0.231 |  |
|  | C | A |  |  | 0.207 | 0.161 | 2.91 | 0.088 | 4.34×10^-4^ | 0.190 | 0.186 | 0.11 | 0.738 | 0.055 | 0.188 | 0.208 | 2.22 | 0.137 | 0.390 |
|  | T | A |  |  | 0.235 | 0.148 | 10.17 | 0.001 |  | 0.212 | 0.180 | 5.56 | 0.018 |  | 0.179 | 0.181 | 0.02 | 0.895 |  |
|  | C | G |  |  | 0.041 | 0.069 | 3.58 | 0.059 |  | 0.019 | 0.026 | 1.82 | 0.177 |  | 0.021 | 0.017 | 0.78 | 0.377 |  |
|  | T | G |  |  | 0.517 | 0.622 | 9.41 | 0.002 |  | 0.579 | 0.609 | 3.14 | 0.076 |  | 0.612 | 0.594 | 1.15 | 0.285 |  |
|  |  | A | T |  | 0.223 | 0.138 | 10.09 | 0.001 | 2.29×10^-4^ | 0.183 | 0.157 | 3.91 | 0.048 | 0.057 | 0.151 | 0.150 | 0.00 | 0.953 | 0.281 |
|  |  | A | G |  | 0.220 | 0.172 | 3.02 | 0.082 |  | 0.222 | 0.210 | 0.70 | 0.402 |  | 0.219 | 0.241 | 2.45 | 0.118 |  |
|  |  | **G** | **G** |  | **0.557** | **0.690** | **15.83** | **6.93×10^-5^** |  | **0.596** | **0.633** | **5.00** | **0.025** |  | 0.631 | 0.609 | 1.72 | 0.190 |  |
|  |  |  | T | T |  |  |  |  | 0.004 | 0.017 | 0.013 | 0.74 | 0.389 | 0.253 | 0.011 | 0.010 | 0.08 | 0.774 | 0.992 |
|  |  |  | G | T | 0.179 | 0.159 | 0.60 | 0.438 |  | 0.092 | 0.092 | 0.00 | 0.997 |  | 0.103 | 0.104 | 0.02 | 0.890 |  |
|  |  |  | T | C | 0.215 | 0.136 | 8.74 | 0.003 |  | 0.169 | 0.147 | 3.08 | 0.079 |  | 0.145 | 0.145 | 0.00 | 0.985 |  |
|  |  |  | G | C | 0.606 | 0.705 | 9.01 | 0.003 |  | 0.723 | 0.748 | 2.86 | 0.091 |  | 0.742 | 0.741 | 0.00 | 0.964 |  |
| C | C | A |  |  | 0.210 | 0.158 | 3.74 | 0.053 | 2.96×10^-4^ | 0.192 | 0.189 | 0.06 | 0.801 | 0.043 | 0.190 | 0.210 | 2.07 | 0.150 | 0.351 |
| **T** | **T** | **A** |  |  | **0.227** | **0.147** | **8.72** | **0.003** |  | **0.208** | **0.173** | **6.72** | **0.010** |  | 0.173 | 0.177 | 0.09 | 0.763 |  |
| C | C | G |  |  | 0.038 | 0.073 | 5.58 | 0.018 |  | 0.019 | 0.024 | 1.13 | 0.288 |  | 0.021 | 0.016 | 1.07 | 0.302 |  |
| C | T | G |  |  | 0.525 | 0.622 | 8.01 | 0.005 |  | 0.581 | 0.614 | 3.89 | 0.049 |  | 0.616 | 0.597 | 1.27 | 0.260 |  |
|  | T | A | T |  | 0.219 | 0.135 | 9.94 | 0.002 | 0.001 | 0.182 | 0.156 | 4.13 | 0.042 | 0.101 | 0.151 | 0.150 | 0.00 | 0.959 | 0.509 |
|  | C | A | G |  | 0.205 | 0.159 | 2.97 | 0.085 |  | 0.192 | 0.187 | 0.15 | 0.701 |  | 0.190 | 0.210 | 2.18 | 0.140 |  |
|  | T | A | G |  | 0.015 | 0.014 | 0.01 | 0.905 |  | 0.031 | 0.025 | 1.02 | 0.312 |  | 0.028 | 0.030 | 0.14 | 0.709 |  |
|  | C | G | G |  | 0.039 | 0.070 | 4.21 | 0.04 |  | 0.019 | 0.026 | 1.97 | 0.161 |  | 0.020 | 0.016 | 1.02 | 0.312 |  |
|  | T | G | G |  | 0.521 | 0.621 | 8.64 | 0.003 |  | 0.577 | 0.607 | 3.18 | 0.075 |  | 0.611 | 0.594 | 1.05 | 0.306 |  |
|  |  | A | T | T | 0.013 | 0.008 | 0.46 | 0.499 | 0.004 | 0.014 | 0.011 | 0.45 | 0.502 | 0.272 |  |  |  |  | 0.485 |
|  |  | A | G | T | 0.107 | 0.072 | 3.08 | 0.079 |  | 0.068 | 0.062 | 0.55 | 0.457 |  | 0.065 | 0.074 | 0.99 | 0.319 |  |
|  |  | G | G | T | 0.069 | 0.084 | 0.62 | 0.430 |  | 0.026 | 0.031 | 0.94 | 0.332 |  | 0.039 | 0.032 | 1.27 | 0.259 |  |
|  |  | A | T | C | 0.209 | 0.130 | 9.26 | 0.002 |  | 0.168 | 0.145 | 3.38 | 0.066 |  | 0.143 | 0.143 | 0.00 | 0.948 |  |
|  |  | A | G | C | 0.113 | 0.101 | 0.35 | 0.553 |  | 0.154 | 0.148 | 0.20 | 0.657 |  | 0.155 | 0.168 | 1.13 | 0.289 |  |
|  |  | G | G | C | 0.489 | 0.606 | 11.88 | 5.68×10^-4^ |  | 0.570 | 0.601 | 3.54 | 0.060 |  | 0.599 | 0.583 | 0.87 | 0.351 |  |

**Additional file 3b**

| SNP* | | | | | **Ashkenazi Jewish** | | | | | **NINDS** | | | | | **CIDR/Pankratz et al 2009** | | | | |
| --- | --- | --- | --- | --- | --- | --- | --- | --- | --- | --- | --- | --- | --- | --- | --- | --- | --- | --- | --- |
| 1 | 2 | 3 | 4 | 5 | **Freq. Case** | **Freq. Control** | **χ^2^** | **P** | **Global**  **P** | **Freq. Case** | **Freq. Control** | **χ^2^** | **P** | **Global P** | **Freq. Case** | **Freq. Control** | **χ^2^** | **P** | **Global P** |
| T | G |  |  |  | 0.167 | 0.177 | 0.16 | 0.689 | 0.040 | 0.153 | 0.163 | 0.60 | 0.438 | 0.272 | 0.159 | 0.144 | 1.45 | 0.229 | 0.380 |
| T | A |  |  |  | 0.208 | 0.275 | 5.40 | 0.020 |  | 0.255 | 0.272 | 1.36 | 0.244 |  | 0.259 | 0.274 | 0.97 | 0.326 |  |
| C | A |  |  |  | 0.626 | 0.548 | 5.35 | 0.021 |  | 0.592 | 0.565 | 2.60 | 0.107 |  | 0.582 | 0.582 | 0.00 | 0.996 |  |
|  | **A** | **A** |  |  | **0.132** | **0.236** | **16.21** | **5.67×10^-5^** | 1.40×10^-4^ | 0.182 | 0.208 | 3.70 | 0.054 | 0.069 | **0.185** | **0.214** | **4.39** | **0.036** | 0.083 |
|  | G | G |  |  | 0.167 | 0.177 | 0.14 | 0.708 |  | 0.152 | 0.162 | 0.73 | 0.394 |  | 0.159 | 0.144 | 1.42 | 0.234 |  |
|  | A | G |  |  | 0.701 | 0.587 | 12.28 | 4.57×10^-4^ |  | 0.667 | 0.630 | 5.03 | 0.025 |  | 0.656 | 0.642 | 0.74 | 0.390 |  |
|  |  | A | G |  | 0.131 | 0.236 | 16.42 | 5.09×10^-5^ | 2.36×10^-4^ | 0.180 | 0.209 | 4.58 | 0.032 | 0.100 | 0.182 | 0.212 | 4.88 | 0.027 | 0.086 |
|  |  | G | G |  | 0.081 | 0.062 | 1.11 | 0.293 |  | 0.107 | 0.102 | 0.30 | 0.587 |  | 0.112 | 0.110 | 0.05 | 0.822 |  |
|  |  | G | A |  | 0.788 | 0.702 | 8.54 | 0.003 |  | 0.713 | 0.690 | 2.20 | 0.138 |  | 0.706 | 0.679 | 3.06 | 0.080 |  |
|  |  |  | G | G | 0.206 | 0.288 | 7.87 | 0.005 | 0.012 | 0.286 | 0.311 | 2.43 | 0.119 | 0.125 | 0.293 | 0.321 | 3.16 | 0.075 | 0.193 |
|  |  |  | A | G | 0.175 | 0.181 | 0.06 | 0.807 |  | 0.161 | 0.171 | 0.65 | 0.419 |  | 0.165 | 0.154 | 0.73 | 0.392 |  |
|  |  |  | A | A | 0.619 | 0.530 | 6.85 | 0.009 |  | 0.553 | 0.518 | 4.13 | 0.042 |  | 0.542 | 0.525 | 1.03 | 0.310 |  |
| T | A | A |  |  | 0.129 | 0.214 | 11.29 | 7.80×10^-4^ | 1.29×10^-4^ | 0.143 | 0.169 | 4.44 | 0.035 | 0.206 | 0.142 | 0.162 | 2.77 | 0.096 | 0.273 |
| C | A | A |  |  | 0.002 | 0.022 | 9.00 | 0.003 |  | 0.039 | 0.039 | 0.01 | 0.930 |  | 0.043 | 0.051 | 1.24 | 0.265 |  |
| T | G | G |  |  | 0.166 | 0.177 | 0.18 | 0.671 |  | 0.153 | 0.162 | 0.50 | 0.478 |  | 0.159 | 0.144 | 1.48 | 0.223 |  |
| T | A | G |  |  | 0.078 | 0.062 | 0.88 | 0.348 |  | 0.112 | 0.103 | 0.67 | 0.415 |  | 0.118 | 0.112 | 0.29 | 0.589 |  |
| C | A | G |  |  | 0.625 | 0.525 | 8.75 | 0.003 |  | 0.554 | 0.527 | 2.48 | 0.115 |  | 0.539 | 0.531 | 0.20 | 0.653 |  |
|  | **A** | **A** | **G** |  | **0.131** | **0.236** | **16.42** | **5.09×10^-5^** | 4.27×10^-4^ | **0.182** | **0.209** | **3.97** | **0.046** | 0.137 | **0.185** | **0.214** | **4.47** | **0.035** | 0.167 |
|  | A | G | G |  | 0.081 | 0.062 | 1.11 | 0.293 |  | 0.107 | 0.102 | 0.24 | 0.622 |  | 0.112 | 0.110 | 0.04 | 0.846 |  |
|  | G | G | A |  | 0.168 | 0.177 | 0.13 | 0.714 |  | 0.153 | 0.163 | 0.63 | 0.427 |  | 0.159 | 0.144 | 1.43 | 0.231 |  |
|  | A | G | A |  | 0.621 | 0.525 | 8.03 | 0.005 |  | 0.558 | 0.526 | 3.47 | 0.063 |  | 0.545 | 0.533 | 0.50 | 0.478 |  |
|  |  | A | G | G | 0.125 | 0.226 | 15.47 | 8.37×10^-5^ | 7.74×10^-4^ | 0.182 | 0.209 | 3.90 | 0.048 | 0.144 | 0.184 | 0.214 | 4.66 | 0.031 | 0.190 |
|  |  | G | G | G | 0.081 | 0.063 | 1.05 | 0.305 |  | 0.106 | 0.102 | 0.18 | 0.671 |  | 0.111 | 0.109 | 0.02 | 0.891 |  |
|  |  | G | A | G | 0.175 | 0.182 | 0.07 | 0.794 |  | 0.161 | 0.171 | 0.58 | 0.446 |  | 0.164 | 0.155 | 0.51 | 0.475 |  |
|  |  | G | A | A | 0.619 | 0.530 | 6.87 | 0.009 |  | 0.551 | 0.519 | 3.51 | 0.061 |  | 0.541 | 0.522 | 1.25 | 0.264 |  |

**Additional file 3c**

| SNP* | | | | | **Ashkenazi Jewish** | | | | | **NINDS** | | | | | **CIDR/Pankratz et al 2009** | | | | |
| --- | --- | --- | --- | --- | --- | --- | --- | --- | --- | --- | --- | --- | --- | --- | --- | --- | --- | --- | --- |
| 1 | 2 | 3 | 4 | 5 | **Freq. Case** | **Freq. Control** | **χ^2^** | **P** | **Global**  **P** | **Freq. Case** | **Freq. Control** | **χ^2^** | **P** | **Global P** | **Freq. Case** | **Freq. Control** | **χ^2^** | **P** | **Global P** |
| G | T |  |  |  | 0.287 | 0.353 | 4.39 | 0.036 | 0.013 | 0.298 | 0.318 | 1.52 | 0.218 | 0.107 | 0.314 | 0.329 | 0.88 | 0.347 | 0.642 |
| G | G |  |  |  | 0.157 | 0.192 | 1.82 | 0.178 |  | 0.114 | 0.129 | 1.86 | 0.173 |  | 0.115 | 0.112 | 0.09 | 0.770 |  |
| A | G |  |  |  | 0.556 | 0.455 | 8.76 | 0.003 |  | 0.588 | 0.554 | 4.19 | 0.041 |  | 0.571 | 0.559 | 0.49 | 0.486 |  |
|  | **G** | **A** |  |  | **0.397** | **0.264** | **16.85** | **4.04×10^-5^** | 2.15×10^-4^ | **0.369** | **0.329** | **6.08** | **0.014** | 0.047 | 0.351 | 0.343 | 0.25 | 0.616 | 0.614 |
|  | T | C |  |  | 0.287 | 0.357 | 4.78 | 0.029 |  | 0.297 | 0.318 | 1.83 | 0.176 |  | 0.313 | 0.329 | 0.97 | 0.324 |  |
|  | G | C |  |  | 0.315 | 0.379 | 3.89 | 0.049 |  | 0.334 | 0.353 | 1.36 | 0.244 |  | 0.337 | 0.329 | 0.22 | 0.638 |  |
|  |  | A | A |  | 0.162 | 0.105 | 5.62 | 0.018 | 5.75×10^-4^ | 0.136 | 0.120 | 1.88 | 0.171 | 0.068 | 0.123 | 0.128 | 0.21 | 0.645 | 0.506 |
|  |  | C | A |  | 0.021 | 0.035 | 1.55 | 0.214 |  | 0.036 | 0.032 | 0.36 | 0.550 |  | 0.030 | 0.038 | 1.51 | 0.219 |  |
|  |  | A | G |  | 0.236 | 0.159 | 7.83 | 0.005 |  | 0.235 | 0.210 | 3.10 | 0.078 |  | 0.228 | 0.215 | 0.89 | 0.346 |  |
|  |  | C | G |  | 0.581 | 0.701 | 13.12 | 2.93×10^-4^ |  | 0.594 | 0.639 | 7.13 | 0.008 |  | 0.619 | 0.619 | 0.00 | 0.974 |  |
|  |  |  | G | T | 0.155 | 0.090 | 7.99 | 0.005 | 0.002 | 0.144 | 0.130 | 1.46 | 0.227 | 0.107 | 0.139 | 0.121 | 2.32 | 0.128 | 0.231 |
|  |  |  | A | G | 0.184 | 0.141 | 2.74 | 0.098 |  | 0.171 | 0.152 | 2.23 | 0.135 |  | 0.153 | 0.166 | 1.05 | 0.305 |  |
|  |  |  | G | G | 0.661 | 0.768 | 11.76 | 6.05×10^-4^ |  | 0.685 | 0.718 | 4.46 | 0.035 |  | 0.708 | 0.713 | 0.09 | 0.763 |  |
| **A** | **G** | **A** |  |  | 0.391 | 0.257 | 17.19 | 3.38×10^-5^ | 6.27×10^-4^ | 0.365 | 0.327 | 5.33 | 0.021 | 0.066 | 0.348 | 0.341 | 0.19 | 0.659 | 0.825 |
| G | T | C |  |  | 0.290 | 0.360 | 4.80 | 0.028 |  | 0.298 | 0.319 | 1.70 | 0.192 |  | 0.314 | 0.329 | 0.90 | 0.343 |  |
| G | G | C |  |  | 0.149 | 0.183 | 1.76 | 0.184 |  | 0.108 | 0.126 | 2.87 | 0.090 |  | 0.112 | 0.110 | 0.05 | 0.825 |  |
| A | G | C |  |  | 0.169 | 0.200 | 1.36 | 0.243 |  | 0.229 | 0.228 | 0.01 | 0.913 |  | 0.226 | 0.221 | 0.16 | 0.694 |  |
|  | G | A | A |  | 0.161 | 0.105 | 5.66 | 0.017 | 0.001 | 0.136 | 0.120 | 1.80 | 0.180 | 0.140 | 0.123 | 0.128 | 0.20 | 0.652 | 0.467 |
|  | G | C | A |  | 0.021 | 0.035 | 1.57 | 0.210 |  | 0.035 | 0.032 | 0.33 | 0.567 |  | 0.029 | 0.037 | 1.51 | 0.219 |  |
|  | G | A | G |  | 0.236 | 0.159 | 7.79 | 0.005 |  | 0.234 | 0.209 | 3.06 | 0.080 |  | 0.228 | 0.214 | 0.86 | 0.352 |  |
|  | T | C | G |  | 0.287 | 0.357 | 4.78 | 0.029 |  | 0.296 | 0.318 | 1.91 | 0.167 |  | 0.313 | 0.328 | 0.93 | 0.335 |  |
|  | G | C | G |  | 0.294 | 0.344 | 2.49 | 0.115 |  | 0.299 | 0.321 | 1.93 | 0.165 |  | 0.307 | 0.292 | 0.90 | 0.344 |  |
|  |  | A | G | T | 0.153 | 0.092 | 7.23 | 0.007 | 6.43×10^-4^ | 0.144 | 0.130 | 1.37 | 0.242 | 0.147 | 0.138 | 0.121 | 2.08 | 0.149 | 0.306 |
|  |  | A | A | G | 0.166 | 0.108 | 5.87 | 0.015 |  | 0.135 | 0.119 | 1.84 | 0.175 |  | 0.123 | 0.125 | 0.04 | 0.851 |  |
|  |  | C | A | G | 0.015 | 0.032 | 2.95 | 0.086 |  | 0.036 | 0.032 | 0.32 | 0.571 |  | 0.030 | 0.041 | 2.76 | 0.097 |  |
|  |  | A | G | G | 0.079 | 0.064 | 0.66 | 0.415 |  | 0.090 | 0.080 | 1.21 | 0.272 |  | 0.091 | 0.097 | 0.35 | 0.556 |  |
|  |  | C | G | G | 0.587 | 0.704 | 12.59 | 3.88×10^-4^ |  | 0.595 | 0.638 | 6.76 | 0.009 |  | 0.618 | 0.616 | 0.01 | 0.905 |  |

**Additional file 3d**

| SNP* | | | | | **Ashkenazi Jewish** | | | | | **NINDS** | | | | |
| --- | --- | --- | --- | --- | --- | --- | --- | --- | --- | --- | --- | --- | --- | --- |
| 1 | 2 | 3 | 4 | 5 | **Freq. Case** | **Freq. Control** | **χ^2^** | **P** | **Global P** | **Freq. Case** | **Freq. Control** | **χ^2^** | **P** | **Global P** |
| C | T |  |  |  | 0.156 | 0.257 | 13.66 | 2.19×10^-4^ | 3.05×10^-4^ | 0.168 | 0.204 | 7.15 | 0.007 | 0.013 |
| A | C |  |  |  | 0.042 | 0.059 | 1.37 | 0.243 |  | 0.041 | 0.032 | 2.24 | 0.135 |  |
| C | C |  |  |  | 0.802 | 0.684 | 15.98 | 6.39×10^-5^ |  | 0.791 | 0.765 | 3.32 | 0.068 |  |
|  | T | C |  |  | 0.157 | 0.257 | 13.39 | 2.53×10^-4^ | 3.63×10^-4^ | 0.165 | 0.199 | 6.39 | 0.012 | 0.004 |
|  | C | C |  |  | 0.045 | 0.062 | 1.28 | 0.257 |  | 0.008 | 0.016 | 4.27 | 0.039 |  |
|  | C | T |  |  | 0.798 | 0.681 | 15.58 | 7.92×10^-5^ |  | 0.826 | 0.786 | 9.18 | 0.002 |  |
|  |  | C | G |  | 0.169 | 0.285 | 16.94 | 3.86×10^-5^ | 2.65×10^-4^ | 0.168 | 0.204 | 7.01 | 0.008 | 0.021 |
|  |  | T | G |  | 0.146 | 0.156 | 0.17 | 0.682 |  | 0.063 | 0.054 | 1.32 | 0.251 |  |
|  |  | C | T |  | 0.032 | 0.032 | 0.00 | 0.985 |  |  |  |  |  |  |
|  |  | T | T |  | 0.653 | 0.527 | 14.16 | 1.68×10^-4^ |  | 0.768 | 0.742 | 3.11 | 0.078 |  |
|  |  |  | G | T | 0.177 | 0.261 | 9.24 | 0.002 | 5.68×10^-4^ | 0.086 | 0.092 | 0.32 | 0.569 | 0.387 |
|  |  |  | T | T | 0.268 | 0.264 | 0.01 | 0.910 |  | 0.366 | 0.361 | 0.09 | 0.770 |  |
|  |  |  | G | C | 0.139 | 0.177 | 2.33 | 0.127 |  | 0.144 | 0.162 | 2.23 | 0.135 |  |
|  |  |  | T | C | 0.417 | 0.298 | 12.98 | 3.14×10^-4^ |  | 0.404 | 0.385 | 1.31 | 0.253 |  |
| C | T | C |  |  | 0.154 | 0.253 | 13.48 | 2.41×10^-4^ | 2.94×10^-4^ | 0.165 | 0.199 | 6.69 | 0.010 | 0.005 |
| C | C | C |  |  | 0.045 | 0.062 | 1.29 | 0.256 |  | 0.008 | 0.016 | 4.28 | 0.038 |  |
| A | C | T |  |  | 0.043 | 0.060 | 1.38 | 0.240 |  | 0.041 | 0.031 | 2.23 | 0.135 |  |
| C | C | T |  |  | 0.758 | 0.624 | 18.42 | 1.77×10^-5^ |  | 0.786 | 0.754 | 4.97 | 0.026 |  |
|  | T | C | G |  | 0.156 | 0.255 | 13.30 | 2.65×10^-4^ | 8.84×10^-4^ | 0.164 | 0.197 | 6.08 | 0.014 | 0.024 |
|  | C | C | G |  | 0.017 | 0.031 | 1.97 | 0.160 |  |  |  |  |  |  |
|  | C | T | G |  | 0.141 | 0.154 | 0.26 | 0.609 |  | 0.063 | 0.051 | 2.15 | 0.143 |  |
|  | C | C | T |  | 0.029 | 0.032 | 0.07 | 0.790 |  |  |  |  |  |  |
|  | C | T | T |  | 0.658 | 0.529 | 14.79 | 1.20×10^-4^ |  | 0.773 | 0.752 | 2.04 | 0.154 |  |
|  |  | C | G | T | 0.106 | 0.176 | 9.14 | 0.003 | 0.002 | 0.110 | 0.135 | 5.11 | 0.024 | 0.161 |
|  |  | T | G | T | 0.072 | 0.087 | 0.72 | 0.397 |  | 0.035 | 0.030 | 0.80 | 0.370 |  |
|  |  | C | T | T | 0.020 | 0.019 | 0.00 | 0.965 |  |  |  |  |  |  |
|  |  | T | T | T | 0.247 | 0.243 | 0.02 | 0.889 |  | 0.402 | 0.382 | 1.48 | 0.223 |  |
|  |  | C | G | C | 0.064 | 0.109 | 5.82 | 0.016 |  | 0.059 | 0.068 | 1.31 | 0.252 |  |
|  |  | T | G | C | 0.074 | 0.069 | 0.09 | 0.761 |  | 0.028 | 0.024 | 0.47 | 0.491 |  |
|  |  | C | T | C | 0.012 | 0.013 | 0.01 | 0.920 |  |  |  |  |  |  |
|  |  | T | T | C | 0.406 | 0.284 | 13.87 | 1.96×10^-4^ |  | 0.366 | 0.360 | 0.10 | 0.749 |  |
