# Supplementary material for: Genome-Wide association study identifies candidate genes for Parkinson's disease in an Ashkenazi Jewish population
Source: BMC Med Genet. 2011 Aug 3;12:104. doi: 10.1186/1471-2350-12-104 (PMC3166909; doi:10.1186/1471-2350-12-104)
Supplement: Additional file 4 — Single SNP association of the genes reported in other studies: GBA, BST1, SNCA, HLA-DRA and LRRK2. Single SNP association of the genes reported in other studies: GBA, BST1, SNCA, HLA-DRA and LRRK2. F_A: Minor allele freq. of case, F_U: Minor allele freq. of control, OR: odds ratios, 95% CI: 95% confidence interval. [file 1471-2350-12-104-S4.DOC]

**Additional file 4**

|  |  |  |  | **Ashkenazi Jewish** | | | **NINDS** | | | **CIDR/Pankratz et al 2009** | | |
| --- | --- | --- | --- | --- | --- | --- | --- | --- | --- | --- | --- | --- |
| **GENE** | **SNP** | **BP** | **Major /Minor** | **F_A** | **P** | **OR** | **F_A** | **P** | **OR** | **F_A** | **P** | **OR** |
|  |  |  | **Allele** | **F_U** |  | **95%CI** | **F_U** |  | **95%CI** | **F_U** |  | **95%CI** |
| ***GBA*** | rs4072037 | 153428691 | G/A | 0.457 | 0.026 | 0.74 | 0.471 | 0.921 | 0.99 | 0.498 | 0.785 | 1.02 |
| ***1q21*** |  |  |  | 0.534 |  | 0.56-0.96 | 0.472 |  | 0.87-1.14 | 0.494 |  | 0.89-1.17 |
|  | **GBAN370S** | **153451576** | **G/A** | 0.056 | 0.049 | 2.06 |  |  |  |  |  |  |
|  |  |  |  | 0.028 |  | 0.99-4.26 |  |  |  |  |  |  |
|  | rs2049805 | 153461604 | C/T | 0.509 | 0.026 | 1.36 | 0.496 | 0.847 | 0.99 | 0.305 | 0.271 | 1.09 |
|  |  |  |  | 0.433 |  | 1.04-1.78 | 0.499 |  | 0.86-1.16 | 0.288 |  | 0.94-1.07 |
|  | rs2990245 | 153464086 | T/C | 0.513 | 0.015 | 1.39 | 0.495 | 0.735 | 1.02 | 0.288 | 0.232 | 0.91 |
|  |  |  |  | 0.430 |  | 1.07-1.82 | 0.489 |  | 0.90-1.17 | 0.306 |  | 0.79-1.06 |
|  | rs1045253 | 153467859 | A/G | 0.353 | 0.024 | 1.40 | 0.286 | 0.573 | 0.96 | 0.308 | 0.205 | 1.10 |
|  |  |  |  | 0.280 |  | 1.05-1.87 | 0.295 |  | 0.83-1.11 | 0.288 |  | 0.95-1.05 |
|  | rs9628662 | 153472965 | C/A | 0.351 | 0.038 | 1.36 | 0.289 | 0.623 | 0.96 | 0.264 | 0.339 | 1.08 |
|  |  |  |  | 0.284 |  | 1.02-1.82 | 0.296 |  | 0.83-1.12 | 0.249 |  | 0.92-1.08 |
|  | rs3180018 | 153496755 | T/C | 0.271 | 0.034 | 1.41 | 0.254 | 0.752 | 1.03 |  |  |  |
|  |  |  |  | 0.207 |  | 1.03-1.95 | 0.249 |  | 0.88-1.20 |  |  |  |
| ***BST1*** | rs3213710 | 15326419 | C/T | 0.465 | 0.718 | 0.95 | 0.473 | 0.804 | 1.02 | 0.449 | 6.39×10^-4^ | 0.79 |
| ***4p15*** |  |  |  | 0.478 |  | 0.73-1.25 | 0.469 |  | 0.89-1.16 | 0.508 |  | 0.69-0.91 |
|  | rs10001565 | 15331671 | T/C | 0.202 | 0.710 | 1.07 | 0.085 | 0.020 | 0.76 | 0.093 | 0.213 | 0.87 |
|  |  |  |  | 0.191 |  | 0.76-1.50 | 0.108 |  | 0.61-0.96 | 0.105 |  | 0.69-1.09 |
|  | rs12502586 | 15335662 | A/G | 0.056 | 0.027 | 0.56 | 0.105 | 0.014 | 1.34 |  |  |  |
|  |  |  |  | 0.096 |  | 0.34-0.94 | 0.080 |  | 1.06-1.70 |  |  |  |
|  | rs955411 | 15345605 | A/G | 0.095 | 0.381 | 1.24 | 0.130 | 0.737 | 1.04 | 0.114 | 0.019 | 0.79 |
|  |  |  |  | 0.079 |  | 0.77-2.01 | 0.126 |  | 0.85-1.27 | 0.140 |  | 0.64-0.96 |
| ***SNCA*** | rs3857059 | 90894261 | G/A | 0.160 | 0.061 | 1.46 | 0.100 | 0.035 | 1.29 |  |  |  |
| ***4q21*** |  |  |  | 0.115 |  | 0.98-2.18 | 0.080 |  | 1.02-1.63 |  |  |  |
|  | rs2736990 | 90897564 | T/C | 0.450 | 0.826 | 0.97 | 0.469 | 0.005 | 0.82 | 0.470 | 0.001 | 0.79 |
|  |  |  |  | 0.458 |  | 0.74-1.27 | 0.517 |  | 0.72-0.94 | 0.528 |  | 0.69-0.91 |
|  | rs3796661 | 90906530 | T/C | 0.114 | 0.050 | 1.61 | 0.038 | 0.024 | 1.57 |  |  |  |
|  |  |  |  | 0.073 |  | 1-2.61 | 0.024 |  | 1.06-2.34 |  |  |  |
|  | rs356191 | 90907143 | T/C | 0.243 | 0.481 | 0.90 | 0.184 | 0.087 | 0.86 | 0.180 | 0.001 | 0.76 |
|  |  |  |  | 0.264 |  | 0.66-1.22 | 0.207 |  | 0.73-1.02 | 0.225 |  | 0.64-0.90 |
|  | rs356188 | 90910560 | C/T | 0.235 | 0.438 | 0.88 | 0.181 | 0.058 | 0.85 | 0.178 | 0.001 | 0.75 |
|  |  |  |  | 0.258 |  | 0.65-1.21 | 0.207 |  | 0.72-1.01 | 0.224 |  | 0.64-0.89 |
|  | rs3775439 | 90928764 | A/G | 0.216 | 0.701 | 1.07 | 0.156 | 0.021 | 1.26 | 0.143 | 0.195 | 1.14 |
|  |  |  |  | 0.205 |  | 0.77-1.48 | 0.128 |  | 1.04-1.52 | 0.128 |  | 0.94-1.38 |
|  | rs3775442 | 90934254 | A/G | 0.080 | 0.037 | 1.86 |  |  |  |  |  |  |
|  |  |  |  | 0.045 |  | 1.03-3.36 |  |  |  |  |  |  |
|  | rs3889917 | 90935875 | C/T | 0.125 | 0.419 | 0.85 | 0.081 | 0.029 | 1.34 |  |  |  |
|  |  |  |  | 0.143 |  | 0.58-1.26 | 0.061 |  | 1.03-1.75 |  |  |  |
|  | rs2197120 | 90948625 | A/G | 0.243 | 0.541 | 0.91 | 0.184 | 0.079 | 0.86 | 0.180 | 0.001 | 0.76 |
|  |  |  |  | 0.261 |  | 0.67-1.24 | 0.207 |  | 0.73-1.02 | 0.224 |  | 0.64-0.90 |
|  | rs894278 | 90953558 | G/T | 0.092 | 0.113 | 1.52 | 0.074 | 0.368 | 1.13 | 0.076 | 0.003 | 1.53 |
|  |  |  |  | 0.062 |  | 0.9-2.57 | 0.066 |  | 0.87-1.47 | 0.051 |  | 1.16-2.02 |
|  | rs3775461 | 90960826 | G/A | 0.125 | 0.419 | 0.85 | 0.081 | 0.029 | 1.34 |  |  |  |
|  |  |  |  | 0.143 |  | 0.58-1.26 | 0.061 |  | 1.03-1.75 |  |  |  |
|  | rs2583978 | 90969349 | A/C | 0.243 | 0.541 | 0.91 | 0.184 | 0.058 | 0.85 | 0.182 | 0.002 | 0.77 |
|  |  |  |  | 0.261 |  | 0.67-1.24 | 0.210 |  | 0.72-1.01 | 0.225 |  | 0.65-0.90 |
|  | rs2583985 | 90974962 | G/A | 0.218 | 0.811 | 0.96 | 0.303 | 0.804 | 1.02 | 0.320 | 0.012 | 1.21 |
|  |  |  |  | 0.225 |  | 0.7-1.33 | 0.299 |  | 0.88-1.18 | 0.280 |  | 1.04-1.40 |
| ***HLA-DRA*** | rs3129882 | 32517508 | G/A | 0.433 | 0.605 | 0.93 | 0.432 | 0.794 | 1.02 | 0.452 | 0.005 | 1.21 |
| ***6p21*** |  |  |  | 0.449 |  | 0.71-1.22 | 0.427 |  | 0.89-1.17 | 0.405 |  | 1.06-1.39 |
|  | rs9268658 | 32518694 | A/G |  |  |  |  |  |  | 0.449 | 0.017 | 0.85 |
|  |  |  |  |  |  |  |  |  |  | 0.490 |  | 0.74-0.97 |
|  | rs2239804 | 32519501 | G/A | 0.319 | 0.950 | 0.99 | 0.468 | 0.555 | 0.96 | 0.449 | 0.017 | 0.85 |
|  |  |  |  | 0.320 |  | 0.74-1.32 | 0.478 |  | 0.84-1.10 | 0.490 |  | 0.74-0.97 |
|  | rs2239803 | 32519811 | A/G |  |  |  |  |  |  | 0.461 | 0.020 | 0.85 |
|  |  |  |  |  |  |  |  |  |  | 0.501 |  | 0.75-0.98 |
|  | rs4935356 | 32520366 | C/A |  |  |  |  |  |  | 0.449 | 0.016 | 0.85 |
|  |  |  |  |  |  |  |  |  |  | 0.490 |  | 0.74-0.97 |
| ***LRRK2*** | rs11175655 | 38909994 | A/G | 0.088 | 0.535 | 1.17 | 0.143 | 0.032 | 1.24 | 0.136 | 0.355 | 1.10 |
| ***12q12*** |  |  |  | 0.076 |  | 0.71-1.92 | 0.118 |  | 1.02-1.52 | 0.125 |  | 0.90-1.34 |
|  | rs10878246 | 38918366 | G/T | 0.114 | 0.651 | 1.11 | 0.207 | 0.027 | 1.21 | 0.217 | 0.275 | 1.10 |
|  |  |  |  | 0.104 |  | 0.72-1.7 | 0.177 |  | 1.02-1.44 | 0.201 |  | 0.93-1.29 |
|  | rs1907632 | 38936769 | A/G | 0.112 | 0.617 | 1.12 | 0.171 | 0.008 | 1.29 | 0.169 | 0.399 | 1.08 |
|  |  |  |  | 0.101 |  | 0.72-1.73 | 0.138 |  | 1.07-1.55 | 0.159 |  | 0.90-1.30 |
|  | rs2723264 | 38938787 | T/C |  |  |  | 0.177 | 0.002 | 0.77 | 0.197 | 0.745 | 0.97 |
|  |  |  |  |  |  |  | 0.218 |  | 0.65-0.91 | 0.202 |  | 0.82-1.15 |
|  | rs10878278 | 38940255 | A/G | 0.243 | 0.677 | 1.07 | 0.174 | 0.016 | 0.81 |  |  |  |
|  |  |  |  | 0.230 |  | 0.78-1.47 | 0.207 |  | 0.68-0.96 |  |  |  |
|  | rs10878307 | 38958256 | C/T | 0.146 | 0.832 | 0.96 | 0.059 | 0.015 | 0.72 | 0.082 | 0.802 | 1.03 |
|  |  |  |  | 0.152 |  | 0.66-1.4 | 0.080 |  | 0.55-0.94 | 0.080 |  | 0.81-1.32 |
|  | rs11175847 | 38962467 | T/G | 0.437 | 0.090 | 1.27 | 0.495 | 0.014 | 1.18 | 0.491 | 0.657 | 1.03 |
|  |  |  |  | 0.379 |  | 0.96-1.67 | 0.453 |  | 1.04-1.35 | 0.483 |  | 0.90-1.18 |
|  | rs12820920 | 38974348 | C/T | 0.437 | 0.090 | 1.27 | 0.495 | 0.015 | 1.18 | 0.492 | 0.610 | 1.04 |
|  |  |  |  | 0.379 |  | 0.96-1.67 | 0.454 |  | 1.03-1.35 | 0.483 |  | 0.91-1.18 |
|  | rs11564203 | 39010848 | T/C | 0.116 | 0.685 | 1.09 | 0.168 | 0.034 | 1.22 | 0.170 | 0.462 | 1.07 |
|  |  |  |  | 0.107 |  | 0.71-1.68 | 0.142 |  | 1.02-1.47 | 0.161 |  | 0.89-1.28 |
|  | rs11829088 | 39014046 | C/A | 0.118 | 0.624 | 1.11 | 0.169 | 0.031 | 1.23 | 0.171 | 0.383 | 1.08 |
|  |  |  |  | 0.107 |  | 0.73-1.71 | 0.142 |  | 1.02-1.48 | 0.160 |  | 0.90-1.30 |
|  | rs10735934 | 39019167 | G/T | 0.455 | 0.410 | 0.89 | 0.462 | 0.046 | 0.87 | 0.472 | 0.682 | 0.97 |
|  |  |  |  | 0.483 |  | 0.68-1.17 | 0.496 |  | 0.76-1.00 | 0.479 |  | 0.85-1.11 |
|  | **rs34637584** | **39020469** | **A/G** | 0.058 | 1.56×10^-4^ | 7.27 |  |  |  |  |  |  |
|  |  |  |  | 0.008 |  | 2.2-23.98 |  |  |  |  |  |  |
|  | rs10506155 | 39022206 | A/G | 0.288 | 0.769 | 1.05 | 0.306 | 0.003 | 0.81 | 0.320 | 0.481 | 0.95 |
|  |  |  |  | 0.278 |  | 0.78-1.41 | 0.353 |  | 0.70-0.93 | 0.331 |  | 0.82-1.10 |
|  | rs7303525 | 39031042 | C/A | 0.213 | 0.634 | 1.08 | 0.171 | 0.002 | 0.76 |  |  |  |
|  |  |  |  | 0.199 |  | 0.78-1.51 | 0.214 |  | 0.64-0.90 |  |  |  |
|  | rs11564173 | 39036738 | A/G | 0.144 | 0.918 | 0.98 | 0.145 | 0.008 | 1.32 | 0.151 | 0.587 | 1.05 |
|  |  |  |  | 0.147 |  | 0.67-1.43 | 0.114 |  | 1.07-1.61 | 0.144 |  | 0.87-1.27 |
|  | rs10878434 | 39044558 | T/C | 0.261 | 0.649 | 0.93 | 0.202 | 0.006 | 0.80 |  |  |  |
|  |  |  |  | 0.275 |  | 0.69-1.26 | 0.241 |  | 0.68-0.94 |  |  |  |
|  | rs3761863 | 39044919 | A/G | 0.369 | 0.370 | 0.88 | 0.326 | 0.032 | 0.86 | 0.350 | 0.992 | 1.00 |
|  |  |  |  | 0.399 |  | 0.67-1.16 | 0.360 |  | 0.74-0.99 | 0.351 |  | 0.87-1.15 |
|  | rs3886747 | 39048218 | C/T | 0.371 | 0.470 | 0.90 | 0.326 | 0.039 | 0.86 |  |  |  |
|  |  |  |  | 0.396 |  | 0.69-1.19 | 0.359 |  | 0.75-0.99 |  |  |  |
