# Supplementary material for: Genome-Wide association study identifies candidate genes for Parkinson's disease in an Ashkenazi Jewish population
Source: BMC Med Genet. 2011 Aug 3;12:104. doi: 10.1186/1471-2350-12-104 (PMC3166909; doi:10.1186/1471-2350-12-104)
Supplement: Additional file 5 — Single SNP association of the genes reported by the International Parkinson Disease Genomics Consortium: STK39, MCCC1 and LAMP3. Single SNP association of the genes reported by the International Parkinson Disease Genomics Consortium: STK39, MCCC1 and LAMP3. OR: odds ratios, 95% CI: 95% confidence interval [file 1471-2350-12-104-S5.DOC]

**Additional file 5**

| **CHR** | **SNP** | **BP** | **Minor/Major**  **Allele** | **Freq.**  **Case** | **Freq.**  **Control** | **P** | **OR** | **95% CI** | **Gene** |
| --- | --- | --- | --- | --- | --- | --- | --- | --- | --- |
| 2 | rs2166963 | 168708539 | T/C | 0.136 | 0.076 | 0.005 | 1.92 | 1.21-3.05 | *STK39* |
| 2 | rs1850438 | 168710868 | A/G | 0.157 | 0.096 | 0.009 | 1.75 | 1.15-2.68 | *STK39* |
| 2 | rs3769395 | 168712371 | A/G | 0.136 | 0.076 | 0.005 | 1.92 | 1.21-3.05 | *STK39* |
| 2 | rs10170500 | 168717811 | G/A | 0.137 | 0.076 | 0.005 | 1.92 | 1.21-3.06 | *STK39* |
| 2 | rs12478804 | 168720626 | G/A | 0.315 | 0.236 | 0.010 | 1.49 | 1.10-2.02 | *STK39* |
| 2 | rs1850440 | 168720748 | A/G | 0.330 | 0.256 | 0.018 | 1.43 | 1.06-1.93 | *STK39* |
| 2 | rs3754775 | 168724509 | T/C | 0.107 | 0.053 | 0.005 | 2.12 | 1.24-3.62 | *STK39* |
| 2 | rs6740826 | 168735801 | T/C | 0.107 | 0.053 | 0.005 | 2.12 | 1.24-3.62 | *STK39* |
| 2 | rs16855160 | 168798166 | C/T | 0.118 | 0.065 | 0.009 | 1.92 | 1.17-3.16 | *STK39* |
| 3 | rs7640612 | 184225961 | T/A | 0.334 | 0.410 | 0.020 | 0.72 | 0.55-0.95 | *MCCC1* |
| 3 | rs1502762 | 184246274 | T/T | 0.188 | 0.256 | 0.015 | 0.67 | 0.49-0.93 | *MCCC1* |
| 3 | rs13078931 | 184261635 | T/T | 0.233 | 0.295 | 0.037 | 0.72 | 0.53-0.98 | *MCCC1* |
| 3 | rs12493050 | 184347767 | T/T | 0.200 | 0.281 | 0.005 | 0.64 | 0.47-0.88 | *LAMP3* |
| 3 | rs605105 | 184350563 | T/A | 0.151 | 0.202 | 0.049 | 0.70 | 0.50-1.00 | *LAMP3* |
| 3 | rs580116 | 184355140 | T/G | 0.153 | 0.216 | 0.016 | 0.66 | 0.46-0.93 | *LAMP3* |
